# Preclinical toxicity analyses of lentiviral vectors expressing the HIV-1 LTR-specific designer-recombinase Brec1

**DOI:** 10.1371/journal.pone.0298542

**Published:** 2024-03-08

**Authors:** Niklas Beschorner, Paul Künzle, Maike Voges, Ilona Hauber, Daniela Indenbirken, Jacqueline Nakel, Sanamjeet Virdi, Peter Bradtke, Niels Christian Lory, Michael Rothe, Maciej Paszkowski-Rogacz, Frank Buchholz, Adam Grundhoff, Axel Schambach, Christian Thirion, Hans-Willi Mittrücker, Julian Schulze zur Wiesch, Joachim Hauber, Jan Chemnitz

**Affiliations:** 1 Leibniz-Institute of Virology (LIV), Hamburg, Germany; 2 German Center for Infection Research (DZIF), Partner Site Hamburg–Lübeck–Borstel–Riems, Germany; 3 PROVIREX Genome Editing Therapies GmbH, Hamburg, Germany; 4 Institute of Immunology, University Medical Center Hamburg-Eppendorf, Hamburg, Germany; 5 Institute of Experimental Hematology, Hannover Medical School, Hannover, Germany; 6 Medical Systems Biology, UCC, Medical Faculty Carl Gustav Carus, TU Dresden, Dresden, Germany; 7 SIRION Biotech GmbH, Gräfelfing, Germany; 8 Hamburg Center for Translational Immunology (HCTI), University Medical Center Hamburg-Eppendorf, Hamburg, Germany; 9 Infectious Diseases Unit, I. Department of Medicine, University Medical Center Hamburg-Eppendorf, Hamburg, Germany; University Hospital Zurich, SWITZERLAND

## Abstract

Drug-based antiretroviral therapies (ART) efficiently suppress HIV replication in humans, but the virus persists as integrated proviral reservoirs in small numbers of cells. Importantly, ART cannot eliminate HIV from an infected individual, since it does not target the integrated provirus. Therefore, genome editing-based strategies that can inactivate or excise HIV genomes would provide the technology for novel curative therapies. In fact, the HIV-1 LTR-specific designer-recombinase Brec1 has been shown to remove integrated proviruses from infected cells and is highly efficacious on clinical HIV-1 isolates *in vitro* and *in vivo*, suggesting that Brec1 has the potential for clinical development of advanced HIV-1 eradication strategies in people living with HIV. In line with the preparation of a first-in-human advanced therapy medicinal product gene therapy trial, we here present an extensive preclinical evaluation of Brec1 and lentiviral vectors expressing the Brec1 transgene. This included detailed functional analysis of potential genomic off-target sites, assessing vector safety by investigating vector copy number (VCN) and the risk for potential vector-related insertional mutagenesis, as well as analyzing the potential of Brec1 to trigger an undesired strong T cell immune response. In conclusion, the antiviral designer-recombinase Brec1 is shown to lack any detectable cytopathic, genotoxic or T cell-related immunogenic effects, thereby meeting an important precondition for clinical application of the therapeutic lentiviral vector LV-Brec1 in novel HIV-1 curative strategies.

## Introduction

Over the years, successful development and subsequent clinical introduction of antiretroviral therapy (ART) among people living with HIV (PLWH) have changed an almost always fatal disease into a manageable chronic illness [1–3]. However, ART cannot cure HIV infection, because ART does not target the integrated viral DNA (the provirus). Thus, rebound viremia is readily observed when medication is stopped [4]. Moreover, lifelong ART may be associated with potential long-term toxicity, adherence problems, and drug resistance [5–9]. Besides, a substantial number of PLWH on ART still exhibit systemic chronic immune activation and, thus, dysregulated immune function, which possibly leads to cardiovascular disease and other inflammation-triggered conditions [10–12]. Thus, given the limitations of ART, there is a growing need to develop novel therapeutic strategies to control or preferably cure HIV infection [13–15].

Meanwhile, the engineering of an entire toolbox of various designer nucleases and recombinases [16, 17] now makes it possible to target viral pathogens with genome editing strategies [18, 19]. When applied to the life cycle of HIV, virus eradication and, in consequence, a cure in its clinical sense might be achievable in the future [20–22]. This notion is essentially based on the previously described clinical cases of the so-called Berlin, London, Düsseldorf, and New York patients [23–26]. These PLWH also suffered from malignancies such as acute myeloid leukemia (AML) [23, 25, 26] or Hodgkin’s lymphoma [24] and underwent allogeneic hematopoietic stem cell transplantation (HSCT) using adult donor stem cells [23–25] or umbilical cord blood cells [26] with a homozygous mutation for the HIV coreceptor CCR5 (CCR5Δ32/Δ32), thereby conferring resistance to HIV-1 R5 subtypes. Unfortunately, such a therapeutic approach cannot be applied to the majority of PLWH, since HLA-matched hematopoietic stem cell (HSC) or cord blood (CB) donors with a homozygous CCR5Δ32 mutation are relatively rare and, perhaps more critical, allogeneic cell transplantation frequently involves potentially hazardous bone marrow myeloablative or reduced-intensity conditioning regimens [27], as well as levels of immunosuppressive treatment. Therefore, it is currently assumed that a scalable cure for HIV, based on gene therapy, i.e. genome-editing, probably involves the genetic *ex vivo* modification of PLWH-derived CD4^+^ T cells or CD34^+^ HSC, followed by autologous cell transfer, or transplantation [28–30]. In analogy to the isolated cases of HIV cure mentioned above, inactivation of the *ccr5* gene in PLWH-derived cells by engineered designer nucleases, such as zinc-finger nucleases (ZFN), transcription activator-like effector nucleases (TALEN), or the CRISPR/Cas system, currently represents a highly active field of HIV cure research [31–35].

A more direct therapeutic approach targets the integrated proviral DNA with CRISPR/Cas [36–38] or engineered designer recombinases [39–41] to remove HIV from the host cell genome. In particular, the site-specific recombinase (SSR) Brec1 has been shown to excise the provirus in the vast majority of HIV-1 primary isolates with high specificity and nucleotide precision due to the conceptual advantage, that the concerted mode of recombinase action mediated by Brec1 does not create overt double strand breaks [42]. Moreover, Brec1 suppresses viral load *in vitro* and *in vivo* (i.e. in HIV-infected humanized mice) to below the limit of detection (<20 HIV-1 RNA copies/ml) [42]. Based on these data, we are currently preparing an advanced therapy medicinal product (ATMP) first-in-human HSC clinical trial using a self-inactivating (SIN) lentiviral vector for Brec1 expression in PLWH.

Here, we provide preclinical analyses of genotoxicity and immunogenicity of the clinical lentiviral vector LV-Brec1 and its derivatives. Our data demonstrate the absence of measurable toxicities due to Brec1 activity or lentiviral gene transfer. Therefore, LV-Brec1 appears to be a central reagent for developing a future curative therapy based on selective Brec1-mediated HIV genome excision.

## Materials and methods

### Lentiviral vectors and production of viral particles

The construction of the lentiviral SIN vectors has been described in detail previously [41, 42].

VSV-G pseudotyped LV particles were produced by transient cotransfection of 293T cells with split-packaging expression plasmids as described [41]. For titer determination, HEK 293T cells were transduced with increasing volumes of lentiviral particles. At 72 h after transduction, the genomic cell DNA was isolated and the amount of Brec1 sequences in relation to the single copy gene *rpp30* was determined by droplet digital PCR (ddPCR).

For selected experiments, the clinical vector LV-Brec1 was produced by Miltenyi Biotec B.V.& Co.KG (Teterow, Germany) following guidelines of good manufacturing practice (GMP).

### Transduction and infection of cell cultures

For analysis of potential Brec1 off-target sites, 1x10^7^ human PM1 T cells (NIH HIV Research Reagent Program; #ARP-3038) were infected in a 50 ml tube with 1 ml RPMI medium + 2 μg replication-competent HIV-1_BaL_Luc2 [42] + 1 μg/ml polybrene for 3 h, washed with 1x PBS and cultivated for 5 days in 30 ml RPMI in a TC-Flask T75 until luciferase assays measured positive for HIV infection. PM1 cells were split 1:1 and cells were transduced, or not, with LV-Brec1. For this, 1x10^6^ cells were transduced twice in 3 ml RPMI with 1x10^8^ IU/ml LV-Brec1 in the presence of 5 μg/ml μl protamine sulfate. Cells were spinoculated at 600 x g for 60 min at room temperature. Culture supernatants were replaced 4 h after transduction by RPMI medium. Every second week, dead PM1 T cells were removed from the respective cultures by hyperdensity gradient centrifugation. Cultures were maintained for up to 16 weeks and, at various time points, analyzed with respect to viral replication (i.e. luciferase expression using the Luciferase Assay Kit from Promega) and gene (i.e. *tat*, *brec1*) transcription by ddPCR.

Likewise, 1 x 10^6^ Jurkat 1G5 cells/ml (NIH HIV Reagent Program; #ARP-1819) were transduced in 6-well plates by the addition of various amounts of lentiviral particles, followed by spinoculation at 650 x g for 10 min at room temperature.

Transduction of primary Lin^-^ bone marrow cells isolated from C57BL/6 mice was performed as follows: 1 x 10^5^ cells/well were placed in a 24-well plate. The total volume was filled up to 700 μl per well with fresh StemMACS* (StemMACS medium supplemented with 50 ng/ml murine SCF, 20 ng/ml murine IL-3, 100 ng/ml human IL-11, 100 ng/ml human FLT3L, 100 U/ml Penicillin/Streptomycin and 2 mM L-Glutamine) together with various amounts of lentiviral or gamma-retroviral particles and 5 μl/ml LentiBOOST [43] (Sirion Biotech) transduction enhancer and spinoculated at 800 x g for 15 min at room temperature. On the second day, cells were transferred to reaction tubes, centrifuged at 300 x g for 5 min at RT, the supernatant removed, cells picked up in 200 μl of fresh StemMACS* medium, seeded back into the same plate and spiked with VSV-G pseudotyped lentiviral (MOI 400) or gamma-retroviral (MOI 20) particles + 5 μl/ml LentiBOOST (Sirion Biotech) transduction enhancer, made up to 500 μl and spinoculated at 800 x g for 15 min at room temperature. The cells were cultured for 14 days and gradually transferred from StemMACS* to IMDM* medium before further analysis.

Transduction of human primary CD34^+^ PBSC isolated from apheresis blood was performed as follows: Cells were taken in culture and maintained in HSC-Brew medium supplemented with human albumin, HSC-Brew supplement and human cytokines TPO, SCF and FLT3L. After two days of culture 5 x 10^5^ cells per 24-well were transferred. The required amount of LV-Brec1 with 2 mg/ml LentiBOOST (Sirion Biotech) was added to the cells and incubated for 6 h in an incubator at 37°C, 5% CO_2_, and 95% relative humidity. After incubation cells were placed in methylcellulose with cytokines for CFU-C assay.

Transduction and infection of human primary CD4^+^ T cells isolated from Buffy Coats of healthy donors were performed as follows: cells were taken in culture and maintained in RPMI medium supplemented with 10% FCS and 10% IL-2. Cells were cultivated in the presence of CD3/CD28 Dynabeads (Gibco) for stimulation. For infection with HIV-1, 5 μg/ml protamine sulfate (Merck) was used as an enhancer, and spin infection was performed (850 x g, 90 min, 30°C). For transduction with LV-Brec1, LentiBOOST (2 mg/ml; Sirion Biotech) was used, followed by spin infection (850 x g, 60 min, 30°C).

### Colony forming unit (CFU-C) assays

The differentiation potential of transduced PBSC cells was performed with StemMACS HSC-CFU Methylcellulose (Miltenyi Biotec GmbH). For this, 1000 transduced or mock-treated cells were suspended in 1 ml of methylcellulose and seeded into a 3.5 cm diameter cell culture dish (Stemcell Technologies). After incubation at 37°C and 5% CO_2_ for 14 days, various cell colonies were identified and counted.

### Droplet digital PCR

To analyze the number of vector copies per cell, primers and probes were designed to detect different vector genes (Table 1). All primers and probes were supplied by Merck KgaA. Genes were quantified by droplet digital PCR (ddPCR) using the QX200 ddPCR platform from BioRad. Genomic DNA was isolated using QIAmp Blood DNA Kit (Qiagen) according to the manufacturer’s protocol for determining integrated copies per genome. Nucleic acid quantities were determined using a NanoPhotometer® N60 from Implen GmbH. To quantify vector genomes per cell, the concentration of the single copy gene *rpp30* was determined in parallel (PrimePCR ddPCR copy number assay RPP30, HEX, BioRad). The ddPCR mastermix per reaction included the following: 10 μl of 2x ddPCR supermix for probes (no dUTP) (BioRad), 1 μl each of forward and reverse primers (1 μM) as well as the probe (0.25 μM) (Table 1), *rpp30* copy number assay and 0.5 μl Hae III for *brec1* or *tat* and Hind III for *pre* as restriction enzymes for DNA fragmentation. 50 ng of template DNA was added and filled to 20 μl total volume with RNase-free water. A non-template control, lacking DNA was included in all assays.

**Table 1:** Primer/probe sequences for ddPCR.

Gene name	Primer sequence	Probe sequence	Primer melting temperature
*brec1*	Fwd: CACAGTGGAAAGCGTGATGAAC Rvs: ATCGCCATCTTCCAGCAGG	6FAM-CATCCGCAACCTGGACAGCGAAACC-BHQ1	55°C
*pre*	Fwd: GGCTTTCGTTTTCTCCTCCTTG Rvs: TCAGCAAACACAGAGCACAC	6FAM-TGGCCCGTTGTCCGTCAACGTG-BHQ1	55°C
*tat*	Fwd: GGCATCTCCTATGGCAGGAA Rvs: TGCTTTGATAGAGAAACTTGATGAGTCT	6FAM-AGCGACGAAGACCTCCTCAAGGCAGT-BHQ1	60°C

The mastermix was used to generate oil droplets using a QX200 Droplet Generator (BioRad), according to the manufacturer’s instructions. The droplets were transferred to the 96-well twin.tec PCR Plates (Eppendorf), which were heat-sealed with tin foil and placed in a thermal cycler with a 105°C heated lid (BioRad). The amplification profile comprised 1 cycle at 95°C/10 min, 40 cycles at 94°C/30 sec (denaturation), and a corresponding primer melting temperature (Table 1) / 60 sec (hybridization and elongation), followed by 1 cycle at 98°C/10 min and hold at 4°C. Following the cycling reaction, plates were placed into a QX200 ddPCR Plate Reader (BioRad), and droplets were analyzed using QuantaSoft software, version 1.7.4. (BioRad).

For the analysis of gene expression, total cellular RNA was isolated using RNAzol (Sigma-Aldrich) according to the manufacturer’s protocol. DNase treatment was performed using RQ1 DNase (Promega). DNase-treated RNA samples were reverse transcribed with M-MLV reverse transcriptase (Promega) using an oligo-dT primer mix. To quantify expression levels, the expression of the RPL13A housekeeping gene was determined in parallel (PrimePCR™ ddPCR™ Copy Number Assay: RPL13A, Human). The ddPCR mastermix / reaction included the following: 10 μl of 2x ddPCR Supermix for probes (no dUTP) (BioRad), 1 μl each of forward and reverse primers (1 μM) as well as the probe (0.25 μM) (Table 1) and *RPL13A* copy number assay. 200 ng template cDNA was added and filled to 20 μl total volume with RNase-free water. A non-template control, lacking DNA was included in all assays.

Droplet generation, transfer, amplification and analysis were carried out as previously described [42].

### Generation of Jurkat 1G5-Tat reporter cells

Jurkat 1G5 cells were transduced with a lentiviral construct expressing a BFP-2A-Tat sequence from an internal phosphoglycerate kinase (PGK) promoter. The transduced cultures were FACS sorted with respect to BFP expression, resulting in stably transduced polyclonal cell cultures, which subsequently underwent limited dilution cloning. A single cell clone, called Jurkat 1G5-Tat, was selected depending on its growing pattern and FACS profile. Subsequently, a vector copy number (VCN) of 4 was determined by ddPCR, and Tat-induced luciferase activity was confirmed by the Luciferase Assay Kit (Promega) following the manufacturer’s instructions.

### Locus-specific capture next-generation sequencing

Capture sequencing was performed using the SureSelect XT HS Kit according to the manufacturer’s instructions (Agilent). The SureSelect custom capture library was designed and produced by Agilent for windows of approx. 2 kb (see S1 Table); windows centered on the target regions within the human genome (UCSC hg38, GRCh38, December 2013). Overall, 4,155 RNA probes with a total probe size of 45,120 kbp were generated. Sequencing of SureSelect enriched libraries was performed on an Illumina NextSeq500 platform (Illumina) with 2 x 150 bp. For each sample between 4.2 to 10.1 million paired end reads were generated.

Regions outside the captured target intervals–with extensions of 1 kb regions upstream and downstream–were masked in the human genome assembly GRCh38 (ftp://ftp.ensembl.org/) using BEDtools [44]. Captured sequenced reads were then aligned to the masked genome using a bwa-mem command [45]. Alignment files in SAM format were converted to its binary version BAM and were sorted and indexed using samtools [46]. Variant (SNP, INDEL) detection was performed on the resulting BAM files using GATK Haplotypecaller with the ‘minimum-mapping-quality 0’ option, and with Platypus [47]. Reads were extracted from the target intervals in BAM format and complete alignment feature statistics for each interval were obtained by using Alfred [48]. For each interval, average per-base depth was calculated using the samtools [46] “depth” command. Furthermore, alignments for all samples were comprehensively visualized within the interesting regions using integrated genomics viewer [49].

Identifying possible rearrangements within a target interval of interest: SAM flags (values of 81, 161, 97, 145, 65, 129, 113 and 177) of all uniquely mapped paired reads with wrong insert size (a large distance between mates or on different chromosome) were manually quantified and combined into a single measurement for all the flags using samtools “view” command and custom Bash script.

### Whole genome sequencing by next-generation sequencing

To measure proviral HIV-1 DNA sequences by whole genome NGS from genomic DNA was isolated on day 14 (10 days post-transduction) of HIV1 infected primary CD4+ T cells. The library preparation and sequencing were carried out as follows: 600 ng– 1μg of gDNA was mechanically sheared (Covaris) to obtain fragments with an average size of 400 bp. Library preparation was performed using the Kapa HyperPrep Kit (Roche), following the manufacturer’s instructions, using Illumina TruSeq Indexed Adaptors (xGen UDI-UMI Adapters, IDT). No amplification cycles were performed. The resulting library was purified with a 0.7x left side and 0.6x right side SPRI bead (Beckman Coulter) size selection and quantified in a Fragment Analyzer (Agilent). Libraries were equimolarly pooled and sequenced on an Illumina NovaSeq 6000 S4 flowcell with paired-end 150 bp reads to a total of 225 million to 1 billion fragments.

### Genotoxicity assays

*In vitro* immortalization assay (IVIM) was performed as previously described in detail [50]. Animal breeding and maintenance were performed at the institutional animal facility of the Leibniz Institute of Virology (LIV; internal LIV project no.: T-2021-01-25). Only adult, healthy and untreated animals were used according the German Animal Protection Law (§4 Abs.3).

Six weeks old male C57BL/6 mice were anaesthetized using isoflurane and subsequently sacrificed by cervical dislocation for bone marrow isolation. Primary Lin^-^ bone marrow cells were isolated from the femurs of these animals. The cells were transduced twice, either with LV-Brec1 or with the highly mutagenic retroviral vector RSF91.eGFPgPRE (RSF91) [50], or remained non-transduced (negative control). Afterwards, the cells were cultured in stem cell media, which was gradually changed to IMDM supplemented with cytokines. At day 15 post-transduction, genomic DNA (gDNA) was isolated from the respective cultures and subjected to VCN analysis by ddPCR. In addition, total cellular RNA was isolated for microarray-based gene expression analysis (see below). Furthermore, a cell aliquot was also seeded into 96-well plates (100 cells/well) for another 14 days. Subsequently, grown colonies were counted (displaying at least half confluency in the respective well). Replating frequencies were calculated with L-calc.

Microarrays were performed by the Research Core Unit Genomics (RCUG) of Hannover Medical School. Details on microarray grid template 084956_D_F_20170713 are available upon request. 100 ng of total RNA were used to prepare Aminoallyl-UTP-modified (aaUTP) cRNA (Amino Allyl MessageAmp™ II Kit; #AM1753; Thermo Fisher Scientific) applying one round of amplification as directed by the company, except for a twofold downscaling of all reaction volumes. Prior to the reverse transcription reaction, 1 μl of a 1:5000 dilution of Agilent’s One-Color spike-in Kit stock solution (#5188–5282, Agilent Technologies) was added to 100 ng of total RNA of each analyzed sample. The labeling of aaUTP-cRNA was performed using Alexa Fluor 555 Reactive Dye (#A32756; Thermo Fisher Scientific) as recommended in the manual of the Amino Allyl MessageAmp™ II Kit (twofold downscaled reaction volumes). cRNA fragmentation, hybridization, and washing steps were carried out as recommended in the ‘One-Color Microarray-Based Gene Expression Analysis Protocol V5.7’, except that 500 ng of each fluorescently labeled cRNA population were used for hybridization. Slides were scanned using the Agilent Micro Array Scanner G2565CA (pixel resolution 3 μm, bit depth 20). Data extraction was performed with the ‘Feature Extraction Software V10.7.3.1’ using the extraction protocol file ‘GE1_107_Sep09.xml’.

Extracted features were analyzed as described on the SAGA website, using R 3.3.2 and Bioconductor 3.4 [51]. Raw data were log2-transformed, quantile-normalized, and within-array replicates condensed using the R package “limma” [52]. Probe annotations supplied by Agilent for the Whole Mouse Genome Oligo Microarray 4x44K v2 (Design ID 026655) were used. Batch correction between different SAGA assays was performed with the ComBat algorithm of R package “sva” [53]. The results of gene expression analysis were visualized as 2D plots. The “prcomp” function from the R package “stats” was used to perform principal component analysis.

Differentially expressed genes between the respective MOCK control of one assay and the control or test vector transduced samples were calculated with the moderated t-test procedure of the”limma” package with Benjamini-Hochberg multiple testing correction. Differential gene expression sets were ranked in descending order resulting in a pre-ranked list of gene symbols consisting of 15801 genes. The gene lists were subsequently analyzed with the Broad GSEA software using “GSEA-preranked” with the (1000) permutation type set to Gene_set [54].

### Analysis of T cell responses

Female C57BL/6J mice were purchased from a commercial provider and kept at the animal facility of the University Medical Center Hamburg-Eppendorf. Mice were housed under specific pathogen-free conditions in individually ventilated cages with standard food and water ad libitum. After adaptation to the facility for two weeks, mice were inoculated with a VSV-G pseudotyped, self-inactivating and replication-incompetent lentiviral vector encoding either Brec1 or cOVA (non-secreted ovalbumin). Mice received 5–8×10^8^ infecting units (IU) in 100 μl sterile PBS intravenously. Control mice received only PBS. Mice were monitored daily by members of the research staff with training in animal experiments or by animal caretakers of the animal facility. Control of mice occurred according to a catalog of conditions outlined in the approved animal protocol with defined humane endpoints. Monitoring included changes in spontaneous behavior (e.g. reduced mobility, abnormal behavior, reduced grooming) and general health conditions (e.g. changes in body weight, abnormal body posture) as well as impaired wound healing or ulceration at the injection site. Mice were sacrificed at a predefined time point of 28 days using CO_2_ and cervical dislocation. 56 mice in total were used for these experiments. In none of the experiments, animals met the criteria for euthanasia before the time point of analysis.

IFN-γ production was detected using an ELISpot Plus: Mouse IFN-γ (ALP) kit (Mabtech, Cincinnati, OH) following the manufacturer’s instructions. Spleens were pressed through 70 and 40 μm cell strainers to isolate the cells. Erythrocytes were depleted using lysis buffer (155 mM NH_4_Cl, 10 mM KHCO_3_, 10 μM EDTA, pH 7.2). Per well, 2×10^5^ lymphocytes were incubated in 200 μl IMDM containing 10% FCS, glutamine, gentamicin and β-mercaptoethanol in antibody-coated plates. Brec1 peptide pools 1–4 (S2 Table) and the immunodominant peptide ovalbumin peptide (OVA_257-264_) were added as stimuli, each peptide with a concentration of 10^-6^ M. Only medium was used as negative controls. Stimuli were run in duplicates. Cultures with anti-mouse-CD3ε antibody (clone 145 2C11) were used for each mouse as positive controls. After 18 h, plates were washed and developed. Spots were counted using an ELISpot reader.

### Data analysis and statistics

For IVIM assays, Indel frequency and off-target rearrangement analysis by NGS, statistical analysis was performed using Prism version 5.03 software (Graph Pad). The statistical significance was assessed by two-way analysis of variance (ANOVA) followed by a Dunnett’s Multiple Comparison Test or Tukey´s Multiple Comparison Test. P< 0.05 (*), p< 0.01 (**), p< 0.001 (***) and p< 0.0001 (****) were considered statistically significant.

Statistics for SAGA results were performed by using Kruskal-Wallis with Dunn’s correction (*p < 0.05; ***p < 0.001; NS = not significant).

Experiments for analyzing cellular expression, antiviral activity and tolerance studies were reproduced in at least three biological replicates consisting of three technical replicates in each experiment. Mathematical means were derived from the data from the technical replicates of these experiments and total errors of these data were calculated and are shown as error bars.

## Ethics statement

Animal experiments were performed in accordance with the German animal protection law and were approved by the “Behörde für Justiz und Verbraucherschutz” of the City of Hamburg (protocol N088/2020).

## Results

### Lentiviral Brec1 gene transfer

**Fig 1:**
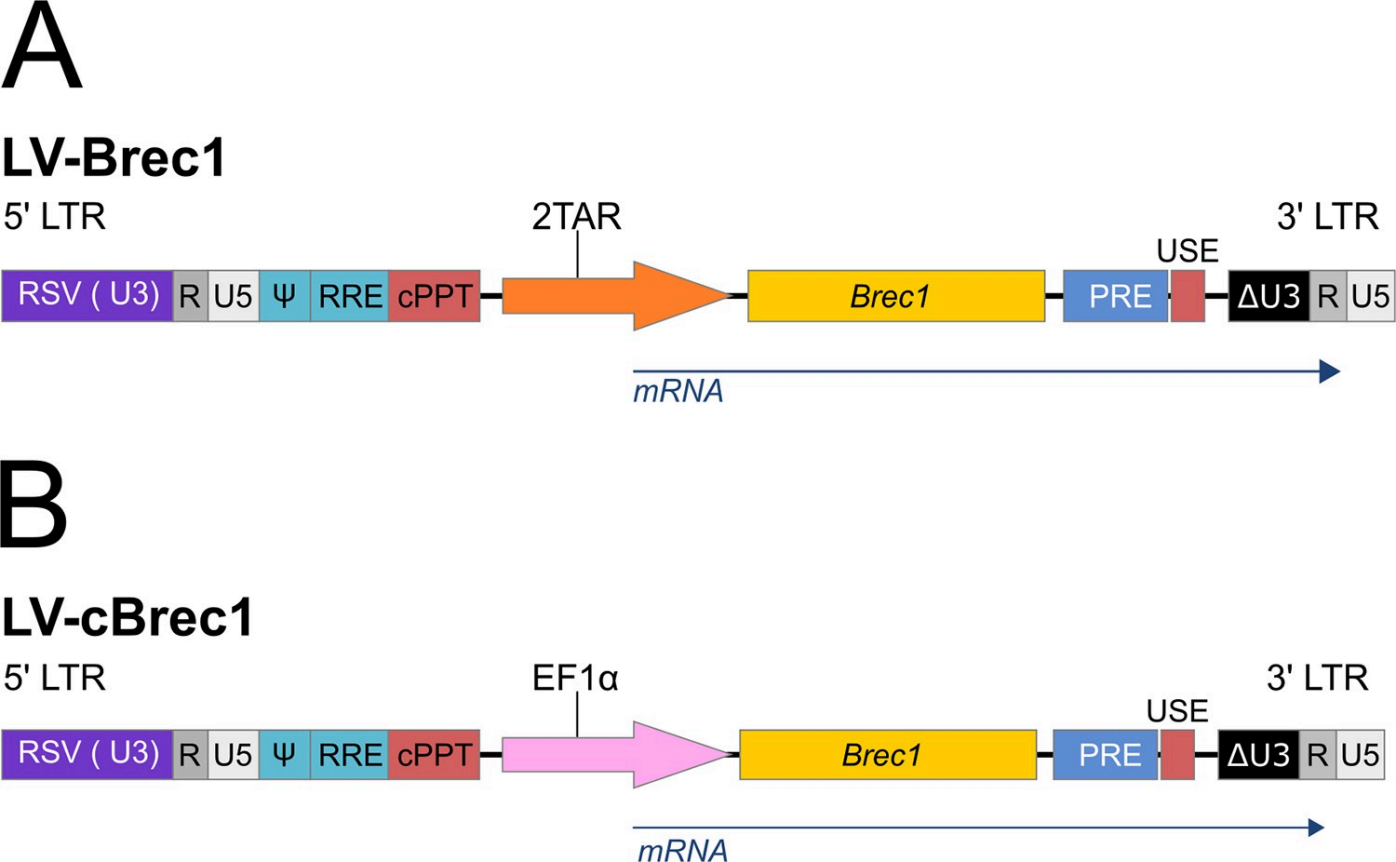
Lentiviral vectors for Brec1 expression. The HIV-derived lentiviral vector backbone contains self-inactivating (SIN) long terminal repeats (LTR: ΔU3, R, U5), a Rev response element (RRE), central polypurine tract (cPPT), posttranscriptional regulatory element derived from woodchuck hepatitis virus (PRE), SV40 upstream polyadenylation enhancer elements (USE). (**A**) In LV-Brec1, the sequence encoding Brec1 recombinase (*Brec1*) is under the control of an HIV-LTR promoter containing two TAR elements (2TAR), allowing conditional (i.e. Tat-dependent) Brec1 expression. (**B**) In LV-cBrec1, constitutive expression of Brec1 is directed by the human EF1α promoter.

Engineered Brec1 recombinase targets a 34-nucleotide sequence, called loxBTR, present in the long terminal repeats (LTR) of the vast majority of clinically relevant HIV-1 primary isolates. Delivery of Brec1 into various target cells was achieved by lentiviral (LV) gene transfer. The self-inactivating (SIN) replication-incompetent vector backbone has been described in detail previously [41]. In the clinical vector LV-Brec1 (Fig 1A), produced following GMP guidelines, the sequence encoding Brec1 was placed under the control of an engineered tandem TAR repeat (2TAR), two *cis*-active target sequences of the HIV-1 Tat *trans*-activator [55], thereby limiting Brec1 expression to only HIV infected cells to ensure a high level of biosafety [42].

To analyze and compare constitutive Brec1 expression, the Tat-inducible 2TAR promoter element in LV-Brec1 was replaced by the human EF1α promoter, resulting in the vector construct LV-cBrec1 (Fig 1B) [42].

### Analysis of potential Brec1 off-target sites

For therapeutic applications, off-target activities of programmable nucleases or recombinases (i.e. designer enzymes) are of major biosafety concerns [56]. For this reason, during non-clinical development, various experimental and computational tools are used for elaborate off-target evaluation of such site-specific designer enzymes [57]. With respect to HIV-1-targeting Brec1, whole genome sequencing analysis of primary CD4^+^ T cells expressing Brec1 previously demonstrated the absence of inadvertent Brec1-mediated human genome alterations [42]. Moreover, *in silico* screening of the human genome for sequences that closely resemble the *bona fide* Brec1 *loxBTR* target site in HIV-1 proviral DNA identified six human genomic sites [42, 58] (Table 2). Importantly, when tested in *E*.*coli*, none of these sites with high sequence similarity to *loxBTR* were recombined by Brec1 [42].

**Table 2:** Potential human genomic Brec1 target sites and their chromosomal location.

Name	Sequence	Genomic coordinates
loxBTR	AACCCACTGCTTAAGCCTCAATAAAGCTTGCCTT	--
HGS1	AA**G**CC**CT**TGCTTAAAAGGATTTAAAG**AA**TG**TT**T**A**	chr1:121169348-121169382(-)
		chr1:143956994-143957028(-)
		chr1:145110955-145110989(+)
		chr1:206187886-206187920(-)
HGS2	AA**ATT**A C/**T** TGCTTATGAAGAAATAAAGC**CA**GC**A**TT	chr8:65603300-65603334(-)
		chr4:138478068-138478102(-)
HGS3	A**T**CC **G**/C AT**A**GCTTATTTAATAATAAAG**T**TTG**TA**T**A**	chr17:20833853-20833887(-)
		chr17:22537726-22537760(-)
HGS4	A**T**CCCACTGCT**G**AATATCCTCTAAAGCTT**T**C**TG**T	Chr6157983492-57983526(-)
		chr6:60734963-60734997(-)
HGS5	**G**AC**G**CA**T**TC**C**TTATTCTTGAA**A**AAAGCTTGC**A**T**A**	chr2:87894679-87894713(-)
		chrX:144143848-144143882(+)
HGS6	**C**AC**AAT**CT**T**CTTACACTGTAGTAAAGCTTGC**T**T**G**	chr9:39631897-39631931(-)
		chr9:42385618-42385652(-)
		chr9:62710515-62710549(+)
		chr9:66745043-66745077(+)
BTR-off3	**CT**CCC**G**CTGCTTACGTGTCTTTAAA**C**C**A**TG**TTCC**	chr1: 159864674 (-).
BTR-off4	**TC**C**AT**AC**A**G**G**TTAGCATGTAATAAA**T**C**A**TG**G**CTT	chr3: 167733225 (-)
BTR-off6	AAC**TGT**CTGCTTAAGGAAATATAA**CT**CTTGC**T**TT	chr7: 125265273 (-)
BTR-off8	AA**AGG**ACTG**G**TTAACACCCCCTAA**TT**C**C**TGCC**CA**	chr12: 103496569 (+)

Nucleotide sequences HGS1-HGS6 display high similarity to *loxBTR*, the sequence targeted in the LTR of HIV-1 isolates, and were previously identified by *in silico* screening of the human genome [42, 58]. Mismatches relative to *loxBTR* (bold) and spacer sequences (gray) are highlighted. Also, the more recently reported potential and non-redundant off-target sites BTR-off3, BTR-off4, BTR-off6, and BTR-off8 [59] were analyzed in this study.

**Fig 2:**
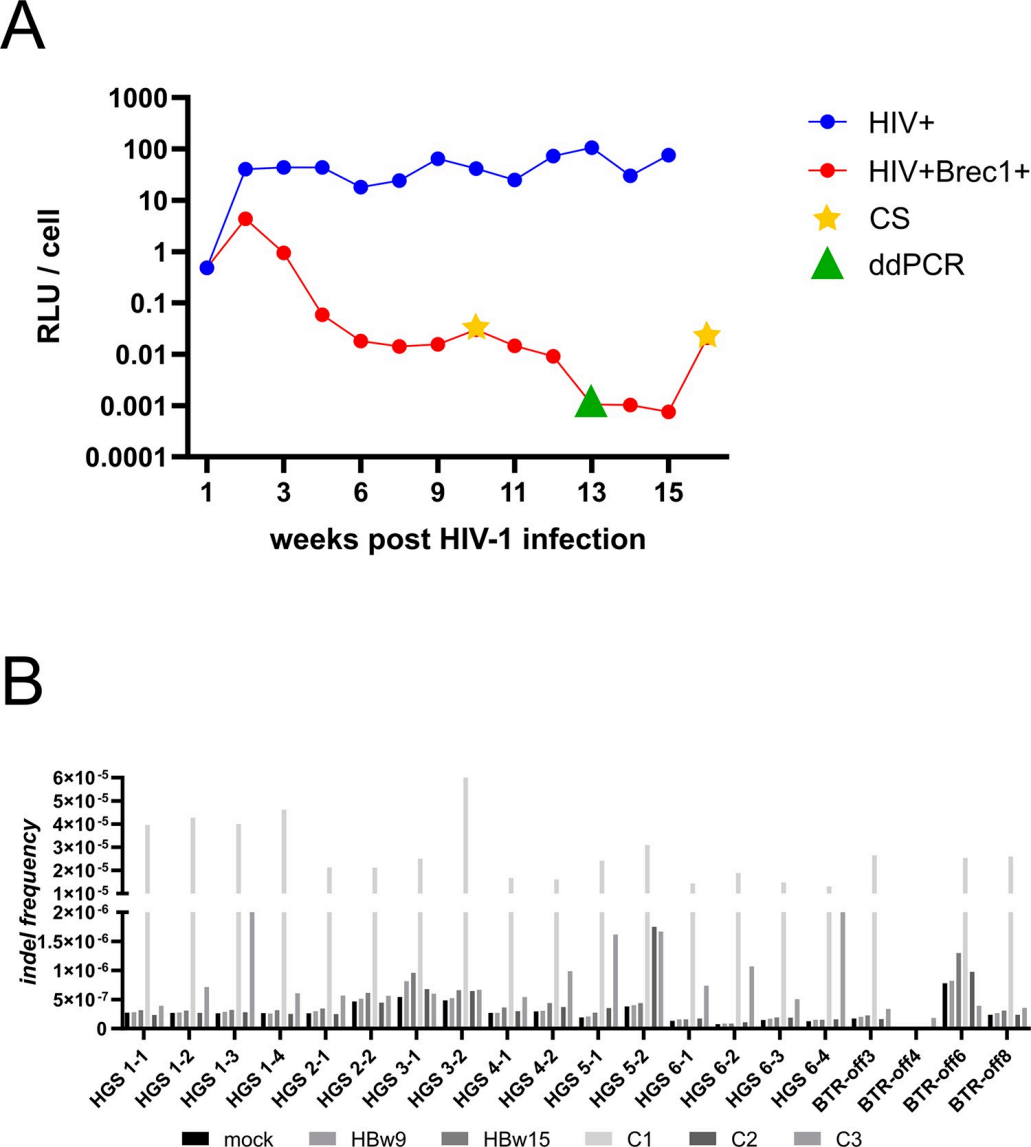
Analysis of potential Brec1 off-target sites. (**A**) Long-term culture of HIV-1-infected and LV-Brec1-transduced or non-transduced PM1 T lymphocytes. A PM1 T cell culture was infected with HIV-1. After a week, half of the culture was transduced with LV-Brec1 (red dots; HIV^+^Brec1^+^), and the other half remained not transduced (blue dots; HIV^+^). HIV-1 replication was measured over time via virus-encoded luciferase activity. Genomic DNA and total cellular RNA were analyzed at the indicated time points by locus-specific capture sequencing (asterisks; CS) or by ddPCR (triangles). The Brec1-transduced culture was reinfected (+HIV) at week 15. (**B**) Visualization of indel frequencies across target regions and samples. Shown is the relative frequency of indels (number of inserted or deleted bases divided by the total number of aligned bases (see Table 3 for absolute values) at the indicated investigated target sites in Brec1-expressing samples (HBw9, HIV^+^Brec1^+^ week9; HBw15, HIV^+^Brec1^+^ week15) or control material (Mock, PM1 gDNA; C1-3, Agilent OneSeq Human Reference DNA).

To analyze the targeting of potential Brec1 genomic sites (Table 2) in more detail and in mammalian cells, we performed locus-specific capture next-generation sequencing (capture sequencing). For this, human PM1 T lymphocytes were infected with a replication-competent CCR5-tropic HIV-1 reporter virus HIV-1_BaL_Luc2, where the *nef* reading frame was substituted by a luciferase (Luc2) encoding sequence [42]. Subsequently, the culture was divided and one aliquot was transduced with LV-Brec1 (Fig 1A). HIV replication was monitored over time by luciferase assays (Fig 2A). At selected time points, levels of HIV-1 *tat* and *brec1* RNA were determined by droplet digital PCR (ddPCR). Chromosomal DNA from the respective cultures was subjected to capture sequencing, covering the potential Brec1 genomic target sites (i.e. off-target sites) HGS 1–6 (Table 2), as well as recently experimentally *in vitro* predicted potential Brec1 pseudosites [59]. Of note, the selected sequences (BTR-off 3, -off 4, -off 6, and -off 8; Table 2) are singular genomic sites and are, therefore, easily accessible to site-specific capture sequencing.

Efficient HIV-1 replication was observed in non-transduced PM1 T cells for 15 weeks (Fig 2A). In sharp contrast, during the same period, virus replication continuously declined to minimal levels in LV-Brec1-transduced cells. At week 15, the Brec1-expressing culture was reinfected with HIV-1. As expected, comparative ddPCR analyses of *brec1* and *tat* RNA levels at week 13 (before reinfection) and week 16 (after reinfection) demonstrated the expected increase in gene expression (*brec1*: three-fold increase; *tat*: four-fold increase), indicating HIV-1 (Tat)-mediated induction of LV-Brec1 expression (Fig 2A).

Total genomic DNA was isolated from the Brec1-expressing cultures at weeks 9 and 15 (HBw9 and HBw15; Fig 2B and Table 3) post-transduction and subjected to capture sequencing. Good coverage overall, with 4.2 to 10.1 million reads for each sample, was generated (Table 3 and S1 Table). By comparison, the number of deleted or inserted bases was very low (on average 137 and 18 bases, respectively). The frequency of bases in indels relative to the total number of aligned bases (indel frequency) per sample and target region was plotted (Fig 2B). If Brec1 expression leads to off-target editing at the investigated sites, we would expect to observe substantially higher indel rates in the Brec1-expressing samples (HBw9, HBw15) relative to the controls (mock and C1-3). However, indel rates were comparable between samples and negative controls even showed a higher indel frequency across all target sites.

**Table 3:** Capture sequencing results of potential Brec1 off-target sites.

*Base counts*																		
target region	aligned^a^						deleted^b^						inserted^c^					
	HB	HB	mock	C1	C2	C3	HB	HB	mock	C1	C2	C3	HB	HB	mock	C1	C2	C3
	w9	w15					w9	w15					w9	w15				
HGS 1–1	4548326	3784595	4956499	3688135	4981772	2254405	149	138	170	123	182	60	42	27	60	23	30	17
HGS 1–2	4601598	3739760	5039794	3726707	4959243	2288310	172	131	161	131	147	68	48	21	60	28	49	20
HGS 1–3	4608666	3790096	5043286	3674385	5027640	2289117	161	126	189	121	171	64	53	27	62	26	72	24
HGS 1–4	4606139	3709664	4975915	3726717	5072955	2296604	172	144	185	147	202	62	35	26	62	25	55	33
HGS 2–1	3725843	3074205	4094412	3056867	4172343	1941663	59	60	97	61	87	37	7	4	7	4	3	2
HGS 2–2	2221531	1740069	2349088	1558140	2307489	1011225	55	58	42	28	34	20	8	4	4	5	1	0
HGS 3–1	1731370	1326001	1846644	1235565	1697073	641450	22	22	50	26	39	24	9	6	0	5	6	1
HGS 3–2	2011797	1566743	2158817	1305740	1703248	656048	944	798	1048	445	741	253	54	31	50	37	74	24
HGS 4–1	3899531	2742979	4084751	2397347	3520852	1477588	99	66	102	38	67	22	6	0	10	2	4	2
HGS 4–2	3573554	2541151	3719768	2048699	3030490	1259992	88	62	122	30	56	29	9	7	12	3	7	10
HGS 5–1	5415037	4670251	5981341	3886518	3064933	2222003	126	85	113	84	62	54	18	24	16	10	5	7
HGS 5–2	2886976	2487092	3296450	906650	611767	538580	48	55	59	25	14	11	8	5	15	3	1	1
HGS 6–1	6864427	6621946	7773694	5990623	6374653	3066335	77	91	91	75	70	56	8	4	3	11	7	2
HGS 6–2	16108551	15395909	18493369	13614889	14459977	7068082	208	211	272	174	196	129	91	70	121	83	113	40
HGS 6–3	6179365	6079825	6921485	5470013	5617025	2799447	65	79	97	74	92	39	3	13	3	7	5	4
HGS 6–4	7308621	7058237	8247916	6296557	6753082	3211654	92	112	103	78	75	36	10	8	7	4	5	5
BTR-off3	5248191	4646423	5806909	4526731	6188347	2680256	118	108	136	115	148	55	8	6	2	5	1	3
BTR-off4	0	0	0	0	0	1466631	0	0	0	0	0	20	0	0	0	0	0	1
BTR-off6	1252933	899228	1403785	749036	1133196	376956	31	12	21	18	28	9	1	2	2	1	3	1
BTR-off8	3781154	3249515	4194254	3042375	4242553	1698422	1081	694	1073	75	84	30	7	2	4	4	1	1

base count samples: HBw9, HIV+Brec1+; HBw15, HIV+ Brec1+ week15; mock, PM1 gDNA; C1-3, reference DNA

a: total number of bases in all reads aligned to the reference target sequence

b,c: number of deleted or inserted bases across all aligned reads

Taken together, the low indel frequency in Brec1 and negative control samples suggests that most (or all) of the observed insertions or deletions represent background noise resulting from misaligned reads, sequencing errors or PCR artifacts introduced during library preparation. The missing reads for BTR-off4 are due to cell line-based sequence variance and the lack of binding of the probes used for capturing. Thus, Brec1 expression did not result in significant genetic alteration of genomic *loxBTR*-like sequences. With respect to the analyzed BTR-off sites, this was further confirmed by next-generation sequencing (NGS) of genomic DNA isolated from Brec1-treated primary CD4^+^ T cells [42]. These data again indicated that these pseudo-sites are indeed not subject to Brec1-mediated recombination (S1 Fig). In summary, no off-target Brec1 activity could be observed in human cells.

### Analysis of a potential rearrangement of off-target sites

**Fig 3:**
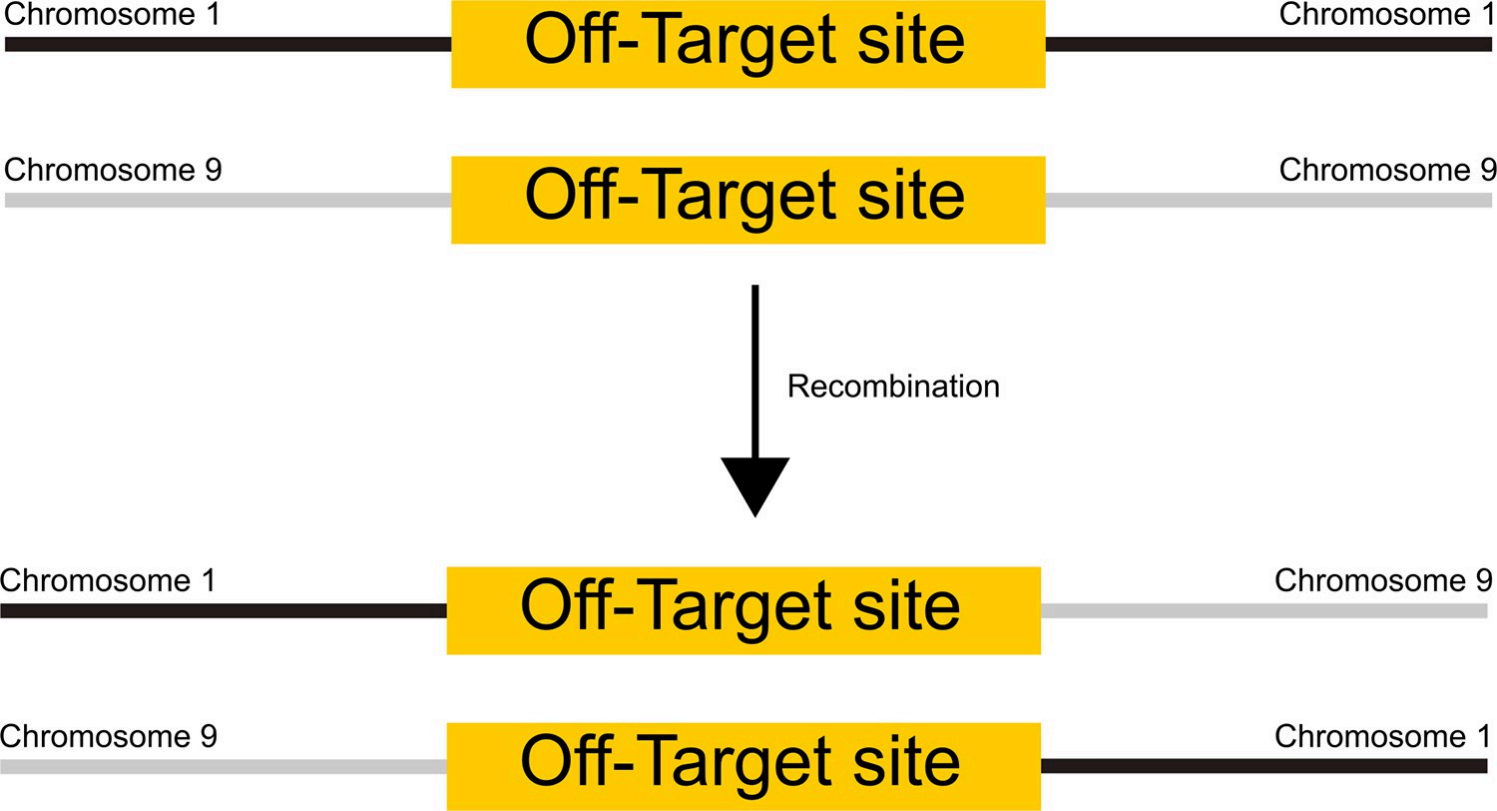
Schematic illustration of a potential rearrangement between two off-target sites on different chromosomes.

For further analysis of potential chromosomal rearrangement triggered by Brec1, the PM1 culture samples were further analyzed. We investigated whether the off-target sites identified in the genome can be recombined by Brec1 in LV-Brec1 transduced cultures in comparison to mock transduced cells. For this purpose, the potential off-target sites were enriched as previously described and sequenced by NGS. A potential rearrangement of two potential off-target sites would lead to a combination of chromosome segments that do not correspond to the reference sequence (Fig 3).

**Fig 4:**
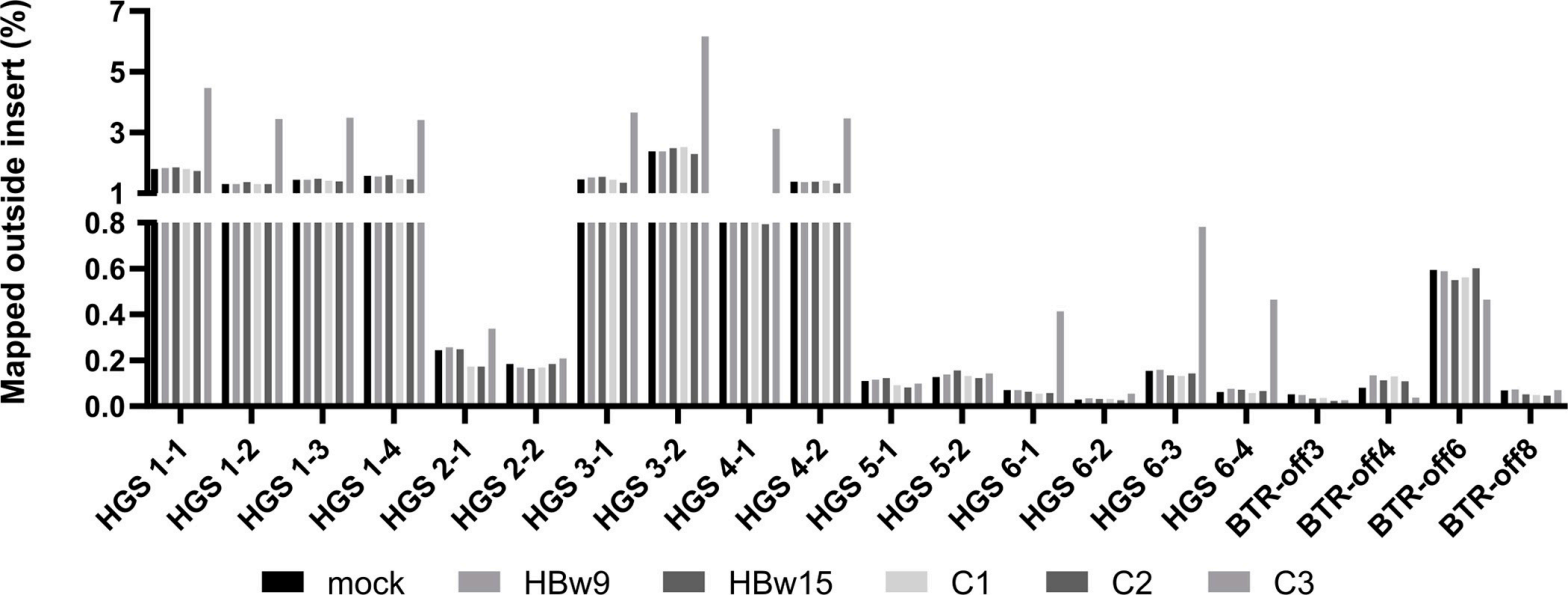
Analysis of reads mapped outside the usual insert size. Regions 1 kb up- and downstream of the target region of 34 bases (in total 2034 bases) were analyzed to check for an increase in sequences that do not correspond to the target region on the same chromosome. For this purpose, the data for the mock-transduced cells were compared to LV-Brec1-transduced cells. Paired t-test of all target sites showed no significant difference between LV-Brec1-transduced and mock-transduced cells at the two time points analyzed.

We examined how many sequences of the target region could not be assigned. By comparing the results of mock-transduced to LV-Brec1-transduced cells by a paired t test, no significant accumulation of unassigned sequences was found at the off-target sites (Fig 4).

### Analysis of a potential rearrangement of off-target sites in HIV^+^ primary CD4^+^ T cells

**Fig 5:**
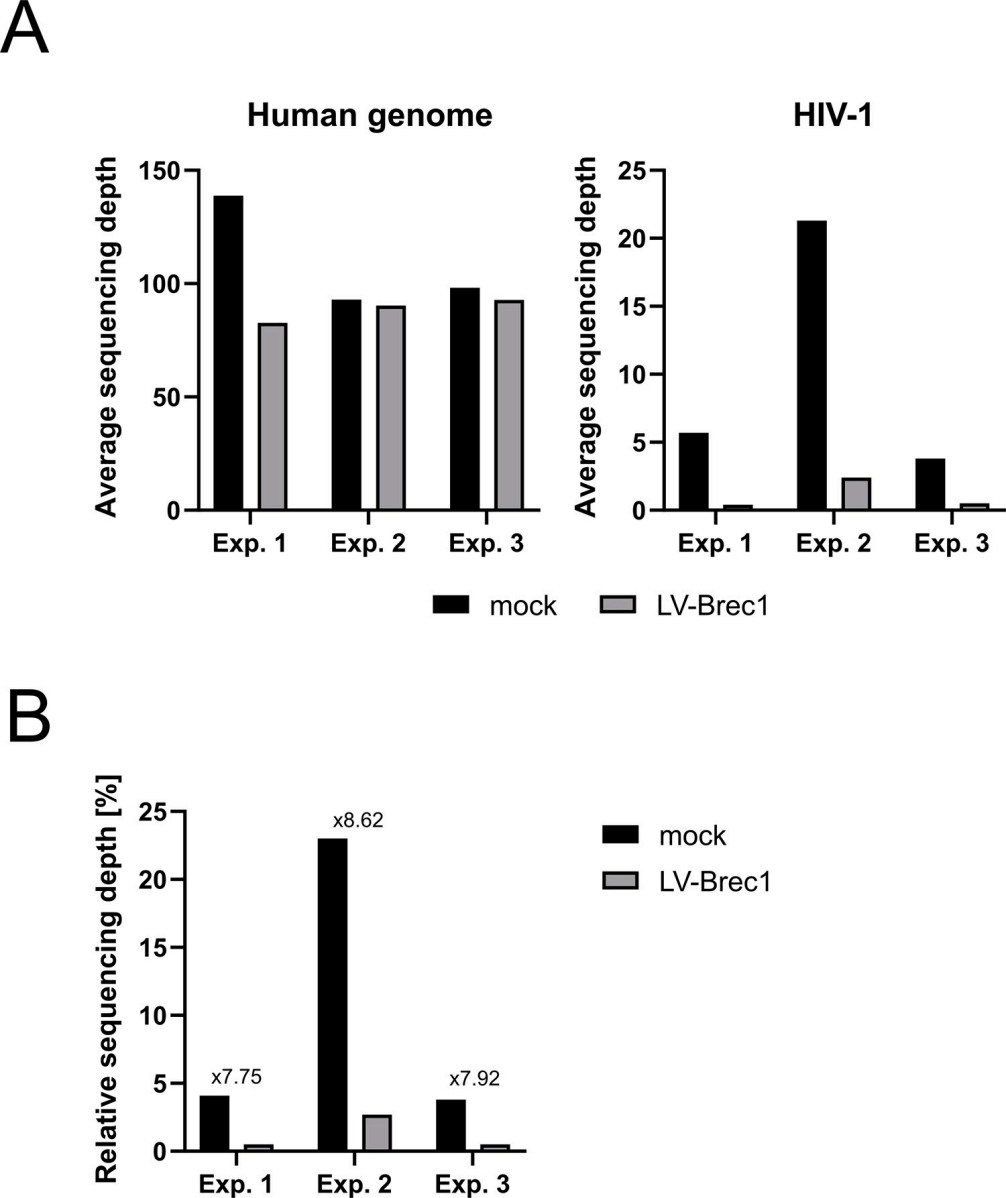
Deep-sequencing analysis. Deep sequencing of genomic DNA isolated at day 14 after the first transduction without LV-Brec1 (mock) and with LV-Brec1 (LV-Brec1) was performed in three replicates (Exp. 1, 2 and 3). (**A**) The left panel shows an average sequencing depth over the human genome. The right panel shows the sequencing depth over the pol locus of HIV. (**B**) Relative HIV1 content in each of the samples, fold reduction of HIV1 of Brec1 treated samples is indicated.

For further analysis of whether chromosomal rearrangement can be induced by Brec1, primary CD4^+^ T cells were infected with HIV-1 in three independent experiments. In each experiment, the infected culture was divided, and one aliquot was subsequently transduced twice with LV-Brec1. At 14 days post-transduction, the genomic DNA of the cultures was isolated, and we determined the virus load and VCN by ddPCR. The gDNA was further analyzed by deep sequencing. Paired-end sequencing reads were mapped to the latest Human Reference Genome (Genome assembly T2T-CHM13v2.0) with an addition of the HIV-1 (HXB2, GenBank K03455.1) complete genome sequence. A comparable sequencing depth of over 80-fold was achieved for all samples. Mock-treated infected cells of the experiment (replicate) 1 (mock) even showed a depth of over 100-fold (Fig 5A). Analysis of the sequencing depth of the HIV pol sequences (HIV-1 genome position 2000–4000) showed a stronger infection in the second experiment. If the number of HIV1 sequences is put in relation, an up to 8.6-fold reduction in HIV1 pol sequences in the LV-Brec1 (LV-Brec1) transduced cultures was observed for all approaches.

**Fig 6:**
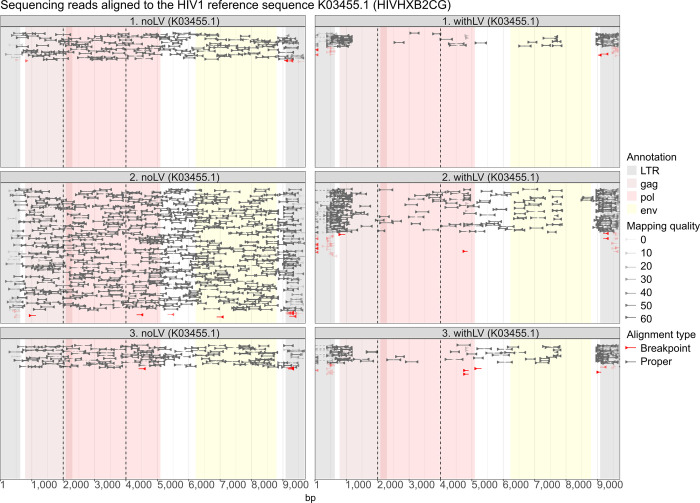
Alignment of NGS reads to the HIV1 genome. The plots show alignments of reads to the HIV genome. Each read is represented by a line starting with an arrow at its 5’ end. Pairs of reads coming from the same fragment are connected by dashed lines. Pairs that are within a reasonable distance and expected orientation (facing each other), are designated as properly aligned and are displayed in grey. All reads whose mates are on different chromosomes are labelled in red. All other reads or pairs, which are aligning discordantly, are displayed in orange. In the case of the HIV alignments, positions of the LTRs, as well as gag, pol and env genes are highlighted.

We also analyzed alignments of the obtained reads to the HIV genome (Fig 6). To confirm the mapping quality, the reads were aligned against any locus on chromosome 1 (S2 Fig). The cells transduced with LV-Brec1 (withLV) showed a clear enrichment of LTR sequences and a loss of HIV-1 sequences, since a single LTR sequence remains in the genome after recombination. Compared to the non-transduced cells (noLV), no increase in discordant reads can be observed.

### Assessment of vector copy number

Vector copy number (VCN) per transduced cell results from the applied vector dosage and is, therefore, of high clinical importance to assess vector safety [60]. It has been shown that target cells with multiple vector insertions bear a higher risk of malignant transformation [61] since increased VCN also increases the potential for insertional mutagenesis [62]e effective VCN that is used in gene therapy approaches.

To analyze VCN and correlating Brec1 expression levels, particularly on the tolerability of varying LV-Brec1-specific VCN, we first generated an appropriate human T cell model. As mentioned above, LV-Brec1 conditionally expresses its payload from a Tat-responsive internal promoter (2TAR; Fig 1A). Therefore, we used Jurkat 1G5 T cells, which contain a chromosomally integrated HIV-1 LTR-luciferase reporter cassette [63]. To provide Tat, thereby mimicking HIV-1 infection, these cells were transduced with a lentiviral vector expressing a BFP-2A-Tat sequence from an internal phosphoglycerate kinase (PGK) promoter. The self-cleaving 2A peptide is derived from a sequence present in picornavirus polyproteins [64] and mediates the co-translational separation of two protein entities that are expressed from a single open reading frame. Thus, the resulting reporter cells, called Jurkat 1G5-Tat, constitutively express the HIV-1 Tat *trans*-activator and thereby activate LTR-driven (e.g. luciferase reporter) gene expression, while the simultaneous expression of BFP allows the identification of successfully transduced cells by FACS (see Materials and Methods). Importantly, Tat will also activate Brec1 expression in LV-Brec1 transduced Jurkat 1G5-Tat cells, thereby providing a cellular assay system for detailed VCN analyses.

**Fig 7:**
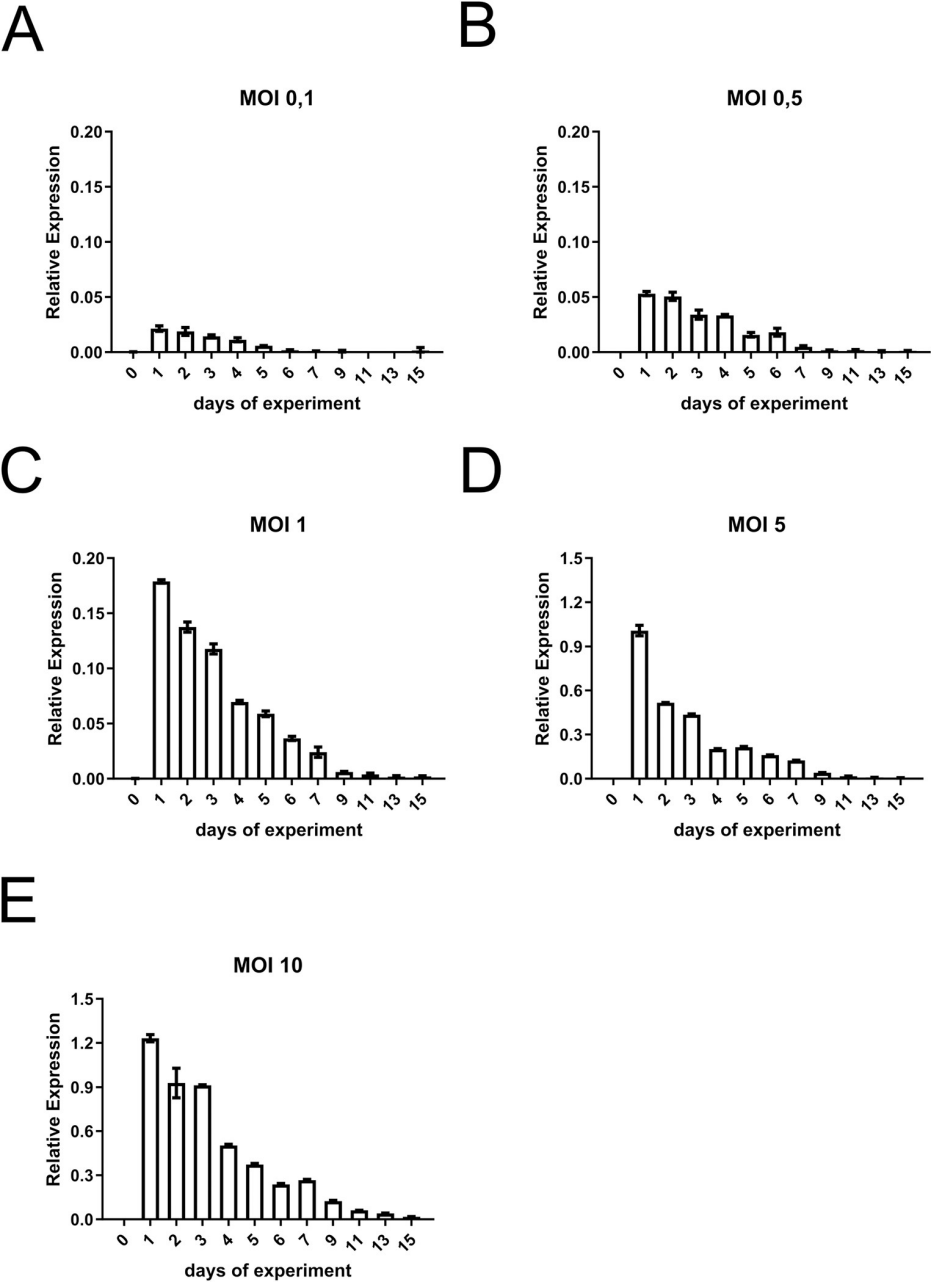
VCN in Jurkat 1G5-Tat cells transduced with LV-Brec1. VCN was determined by ddPCR using genomic DNA isolated at the indicated day (d) after transduction with different MOI (**A-E**). This experimental setup was repeated three times, resulting in a total of three biological replicates with technical triplicates from each experiment, resulting in comparable results. Representative results from one biological replicate with three technical replicates are shown. Total error bars from the technical replicates are shown.

Cultures of the respective Jurkat 1G5-Tat cell clone were transduced with different MOIs of LV-Brec1 (MOI 10, 5, 1, 0.5, 0.1, and 0 as a control; Fig 7). For eight days (days 0–7), as well as on days 9, 11, 13 and 15, the cultures were analyzed with respect to VCN (by ddPCR of genomic DNA), relative Brec1 gene expression (by RT-ddPCR using total cellular RNA; see below), as well as cell viability (by MTT assays; not shown). Meaningful VCN were observed starting with an MOI of 1, which then continuously declined over the time course (Fig 7).

**Fig 8:**
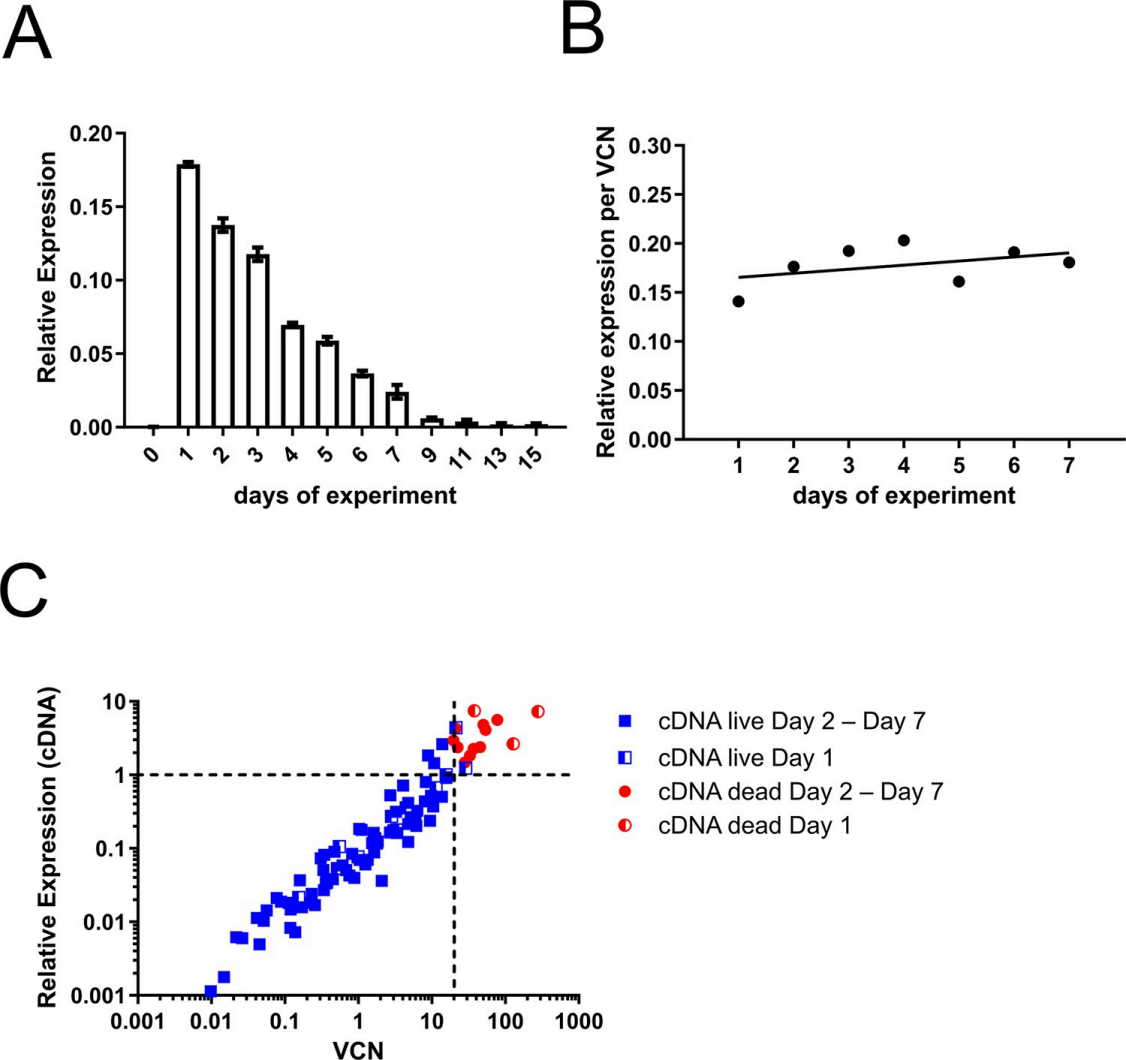
A direct relationship between VCN and relative transgene expression. (**A**) Relative Brec1 expression by LV-Brec1 in Jurkat 1G5-Tat cells at MOI 1. At the indicated time points, RNA samples were reversely transcribed and subjected to ddPCR using Brec1-specific oligonucleotides. These experiments were repeated three times with three technical replicates each. Total error bars are shown. (**B**) Time plotted against the quotient of relative transgene expression per VCN. (**C**) VCN and relative transgene expression data in relation to cytotoxicity. Results from all experiments mentioned before (all MOI, all time points, all biological and all technical replicates) were plotted on logarithmic scales to the base 10. Blue squares depict cells staying viable over the time courses. Red dots mark cells that underwent apoptosis during the experimental time frame. The results for day 1 post-transduction are indicated by blue/white or red/white symbols.

Since the U.S. Food and Drug Administration (FDA) recommends that the VCN for gene therapy products should be <5 [65], we concentrated on an MOI of 1 in the next analyses. VCN decline correlated directly with decreasing levels of relative Brec1 gene expression (i.e. mRNA levels) over the entire time course (Fig 8A). When the quotient of relative gene expression per VCN was plotted over time, no obvious changes in these parameters were observed (Fig 8B), indicating stable transcription and expression of intracellular Brec1-expression cassettes.

Analysis of Brec1 gene expression and VCN data in relation to cytotoxicity (as measured by MTT assays) in the individual cell culture samples also demonstrated a linear correlation of VCN and gene expression, while cytotoxicity occurred particularly at high VCN and corresponding high gene expression (Fig 8C). Furthermore, these data also demonstrated that cells with comparable VCN appear to be characterized by almost identical levels of relative transgene expression, and no saturation or limitation with respect to gene expression was reached in these experiments. Taken together, the combined analyses in Jurkat 1G5-Tat reporter cells indicated that a treatment window for LV-Brec1 exists, which is characterized by <5 vector copies per transduced cell (VCN), while the respective cell cultures stayed viable over the entire experimental time.

### Analysis of vector genotoxicity

Integrating retro- or lentiviral vectors used in HSC gene therapies may trigger leukemogenic malignancies due to insertional mutagenesis [66]. However, the development of viral vectors with a self-inactivating (SIN) LTR design, expressing the transgene from an internal promoter, has reduced this type of severe adverse event as much as possible [67, 68]. Nevertheless, a detailed assessment of potential vector genotoxicity remains an important milestone in the pre-clinical development of novel gene therapeutic approaches. Therefore, we investigated LV-Brec1 toxicity with regard to insertional mutagenesis by *in vitro* immortalization (IVIM) analysis [50] and surrogate assay for genotoxicity assessment (SAGA) [69].

**Fig 9:**
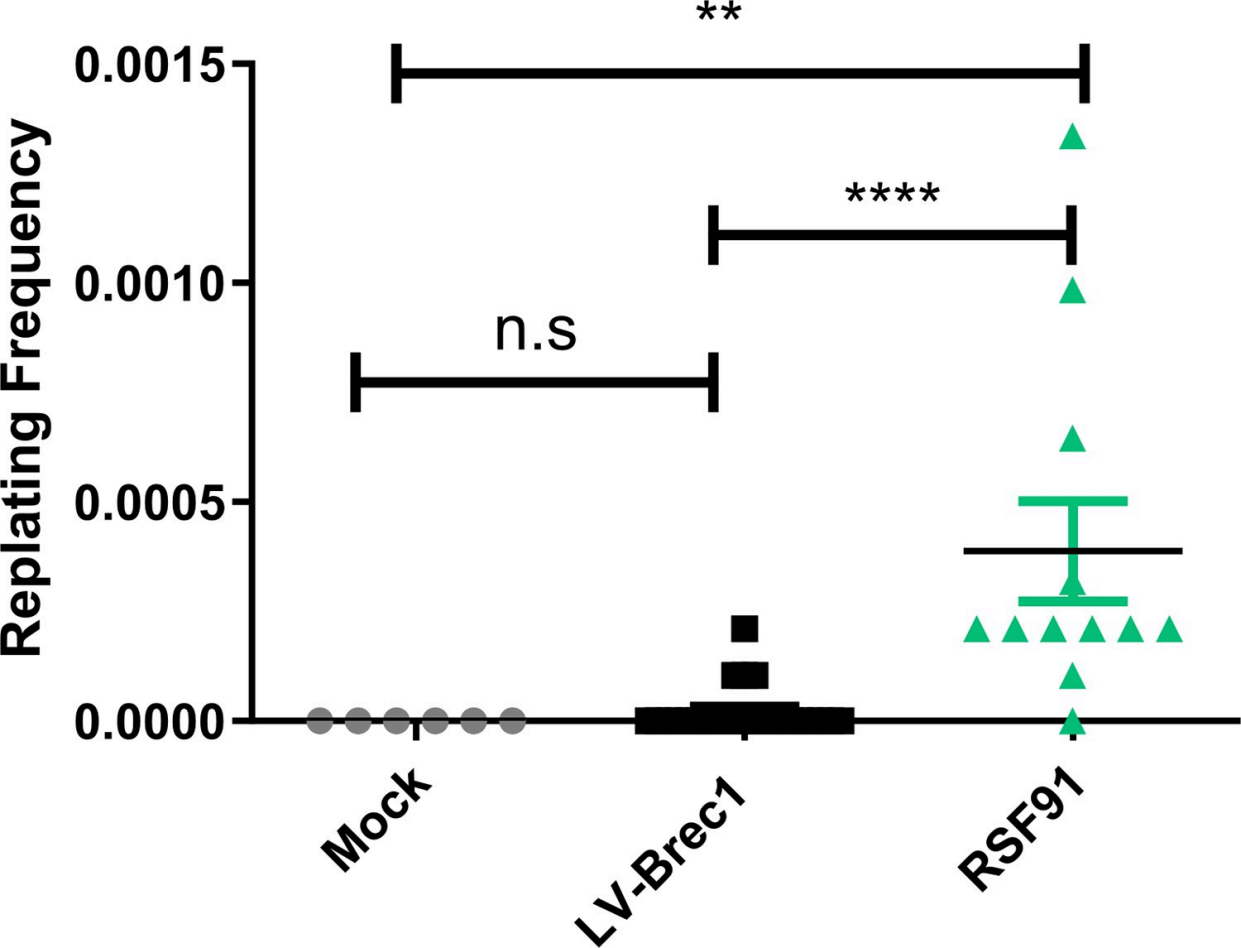
Frequency of clonal growth in transduced murine Lin^-^ bone marrow cells. For IVIM assays, primary Lin^-^ bone marrow cells of C57BL6 mice were transduced twice with LV-Brec1 (black squares) or RSF91 positive control vector (green triangles), or left non-transduced as negative controls (grey, dots). At day 15 post-transduction, limiting dilution was performed in 96-well plates with 100 cells/well. After another 14 days of cultivation, colonies grown in the individual wells (displaying at least half confluency in the respective well) were counted. Replating frequencies were calculated with L-calc and two-way ANOVA, followed by Tukey´s multiple comparison tests for statistical evaluation in Prism.

First, primary Lin^-^ bone marrow cells, isolated from C57BL6 mice, were transduced with GMP-grade LV-Brec1 or with laboratory-grade RSF91, an established gamma-retroviral control vector that has previously been shown to induce genotoxicity in IVIM assays [50]. Non-transduced cells were used as negative controls. At day 15 post-transduction, genomic DNA and total cellular RNA were isolated from the respective cultures to determine transduction efficiency (i.e. VCN) by ddPCR (S3 Fig) and microarray-based gene expression analysis. In addition, a cell aliquot was replated by limiting dilution and cultured for another 14 days for visual inspection by microscopy and counting of grown colonies. A significant difference between the replating frequencies of LV-Brec1 transduced and RSF91-transduced cells were observed (Fig 9). However, the replating frequency of LV-Brec1 transduced cells was not significantly different when compared to mock transduced (i.e. non-transduced) cells.

In addition, the isolated total cellular RNA was analyzed by the more robust and sensitive surrogate assay for genotoxicity assessment (SAGA) [69]. SAGA is based on the dysregulation of genes involved in the immortalization process of murine hematopoietic cells. SAGA development involves machine learning algorithms to obtain a specific SAGA core signature, which distinguishes immortalized from non-immortalized samples. A set of 11 genes (*Zbtb16*, *Itih5*, *Spns2*, *Aff3*, *Sla2*, *Art4*, *Traf4*, *Naip1*, *Slco3a1*, *Frat2*, *Tie1*) can be used to estimate the mutagenic risk by a support vector machine and conventional gene set enrichment analysis (GSEA). Thus, the risk of insertional mutagenesis is reflected by two bioinformatic estimators. First, the test sample is projected into the dataspace of the previously measured positive and negative controls in a principal component plot (not shown). In a second step, the SAGA core set is used to calculate a normalized enrichment score (NES) for the different samples in GSEA [54]. A high NES, as observed for mutagenic vectors such as RSF91, implies an increased mutagenic potential. A low NES in GSEA, together with a projection into the dataspace of non-immortalized samples, argues in favor of a beneficial safety profile of a test vector.

**Fig 10:**
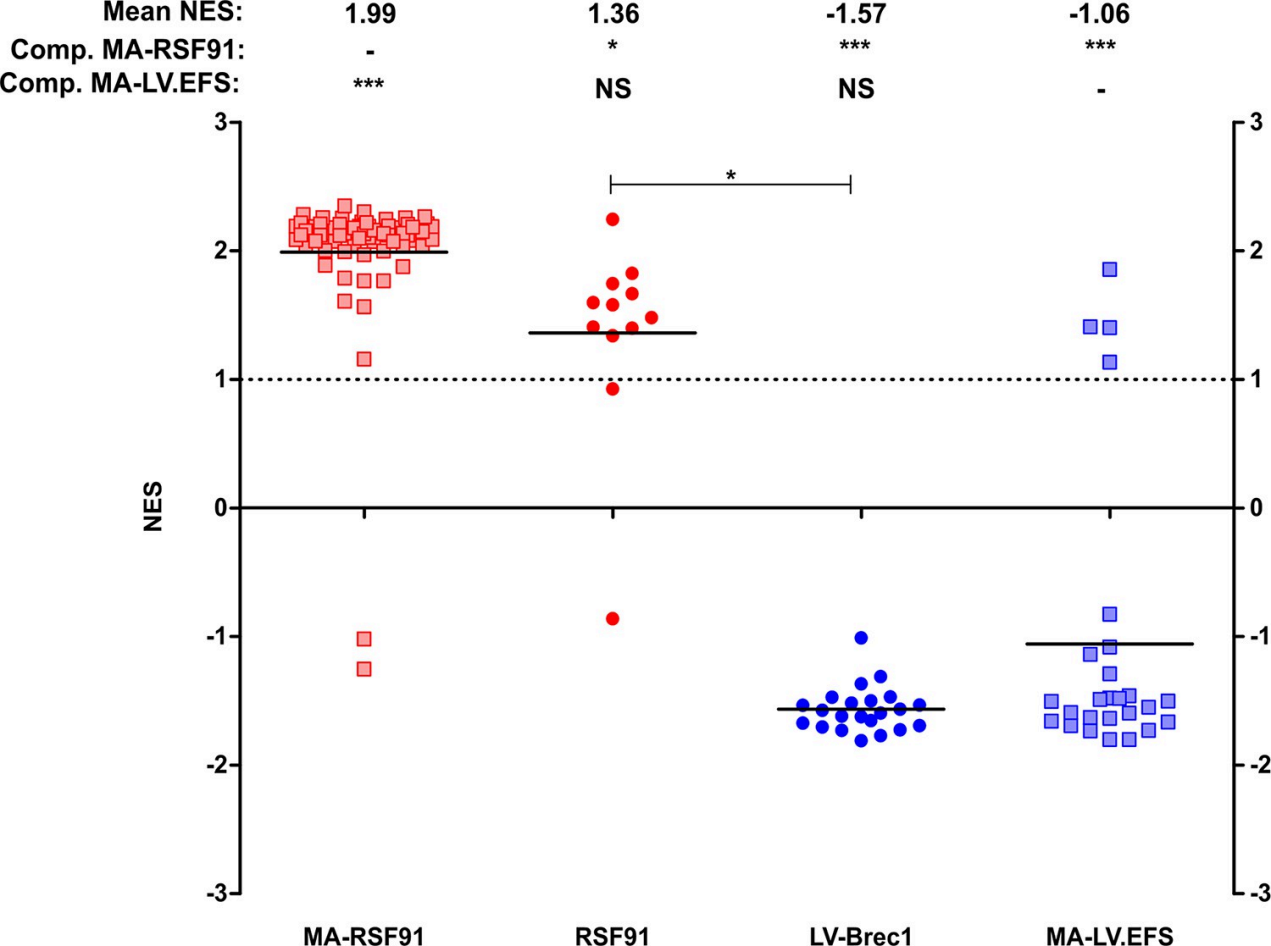
Gene expression analysis by GSEA. A positive normalized enrichment score (NES) indicates an upregulation of an oncogenic core set of genes. Current samples were compared to previously measured positive controls (MA-RSF91) or SIN lentiviral vectors with an internal EFS promoter (MA-LV.EFS) as a negative control. Bars indicate means (also listed above the graph). Statistical comparison to MA-RSF91 by Kruskal-Wallis with Dunn’s correction (*p < 0.05; ***p < 0.001; NS = not significant).

We performed GSEA analysis of LV-Brec1 with respect to the potential upregulation of an oncogenic SAGA core signature (Fig 10 and S3 Table). A normalized enrichment score (NES) above 1 indicates an increased mutagenic risk. NES scores of the current assays were compared to metadata of RSF91 (MA-RSF91) or SIN lentiviral vectors with an internal EFS promoter (MA-LV.EFS) [69].

In total, 10 out 12 RSF91 samples from the assay described below were predicted to be transforming. The mean NES score for RSF91 was 1.36, which was statistically lower than the metadata of this vector. Importantly, all LV-Brec1 samples had a negative NES (mean: -1.57), which is significantly different from the metadata of RSF91 and our current RSF91 samples from the same assay. LV-Brec1 scored even lower than the metadata of a SIN.LV.EFS vector configuration, which is believed to be relatively safe.

Taken together, LV-Brec1 neither induced significant aberrant cell growth by insertional mutagenesis (IVIM assay), nor dysregulation of the SAGA gene set.

### Vector genotoxicity and differentiation potential of transduced CD34^+^ PBSC

**Fig 11:**
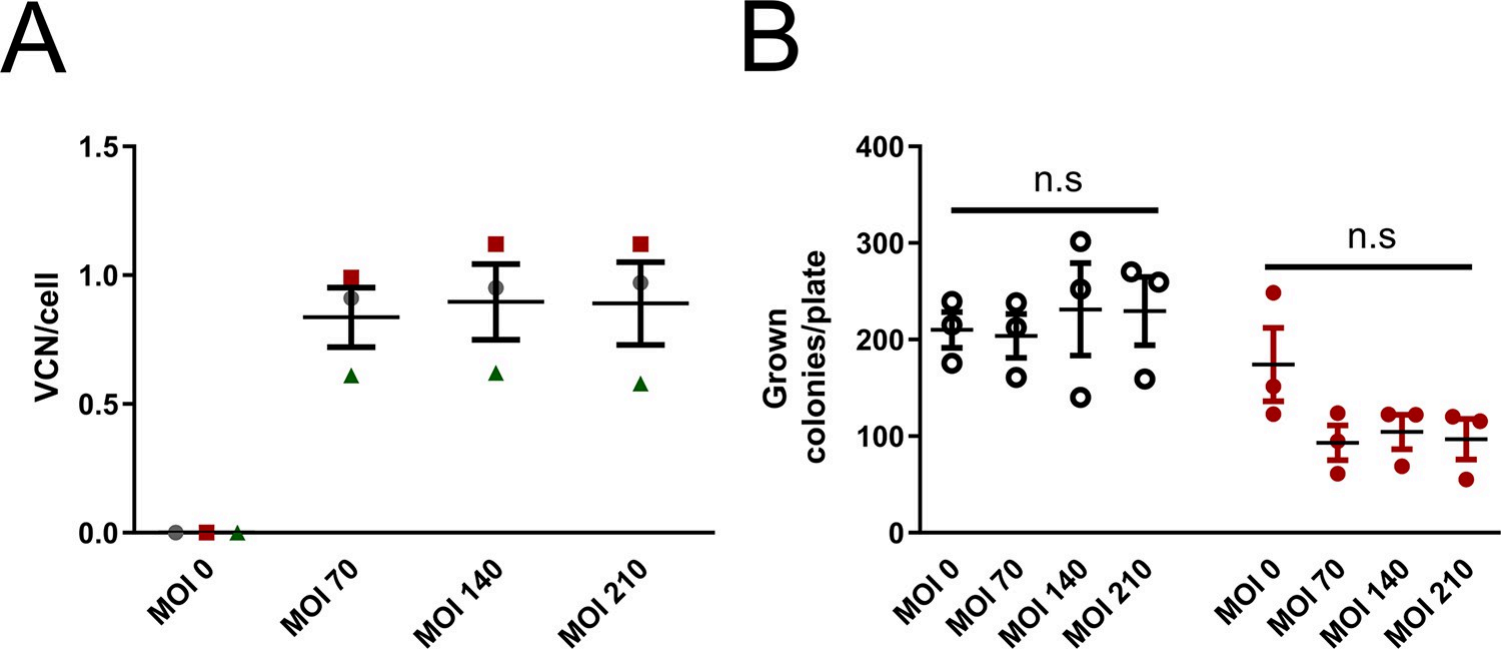
Transduction efficiency and CFU-C assay. (**A**) Human primary CD34+ PBSC were transduced with LV-Brec1 with MOI 0, 70, 140 and 210. One day later, cells were washed, placed in methylcellulose medium containing cytokines and incubated for a further 14 days for differentiation into their progeny cells. Genomic DNA was isolated and VCN was determined via ddPCR. VCN mean values of each assay are plotted (red: Exp1, gray: Exp2, green: Exp3). (**B**) CFU-C colonies were counted and divided into white colonies (ring symbol) and red colonies (red dots). Two-way ANOVA followed by Dunnett´s Multiple Comparison Test was used for statistical evaluation with non-transduced cells (MOI 0) as reference and showed no difference in the differentiation potential.

To further analyze potential vector genotoxicity, we evaluated the influence of LV-Brec1 on the differentiation potential of CD34^+^ peripheral blood stem cells (PBSC) performing CFU-C Assays. PBSC from two different donors were transduced with increasing MOI of LV-Brec1 (Fig 1A) in three independent experiments. After transduction, PBSC were seeded in methylcellulose containing cytokines for 14 days. After incubation, grown colonies were counted and divided into red (CFU-E, BFU-E) and white (CFU-M, CFU-G, CFU-GM, CFU-GEMM) progeny colonies (Fig 11A). Subsequently, genomic DNA was isolated from the respective CFU assay colonies and the number of integrated copies of LV-Brec1 per genome was determined by ddPCR (Fig 11B).

A VCN ranging from 0.6 to 1.1 VCN per cell could be achieved in the case of CFU-C. No significant increase in VCN was observed by doubling the input MOI. As expected, the variation in VCN seems to be donor-dependent (Fig 11).

No difference in clonogenic potential between the three different MOIs in relation to the amount of red or white colonies was observed. Also, there was no difference in the number of grown white colonies (CFU-GM cells) between transduced and mock-transduced cells. However, the red progeny colonies showed a slightly lower number of colonies compared to mock-transduced cells, independent of MOI, but the difference was not statistically significant.

### Analysis of potential Brec1-induced T cell responses

Lentiviral vectors expressing therapeutic transgenes have become a promising gene therapy strategy to treat, for example, inherited malignancies or infectious diseases [70, 71]. However, expression of the therapeutic transgene may trigger undesired T cell responses in the host [72, 73]. We therefore next investigated constitutive lentiviral Brec1 expression in mice and whether this intracellular located Brec1 is able to induce strong T cell and cytokine responses by MHC presentation in vivo.

Female C57BL/6J mice were infected with a lentiviral vector encoding either Brec1 or cOVA (non-secreted ovalbumin; positive control for T cell response). Control mice received only PBS. After 28 days, spleens were collected and analyzed by ELISpot assays.

**Fig 12:**
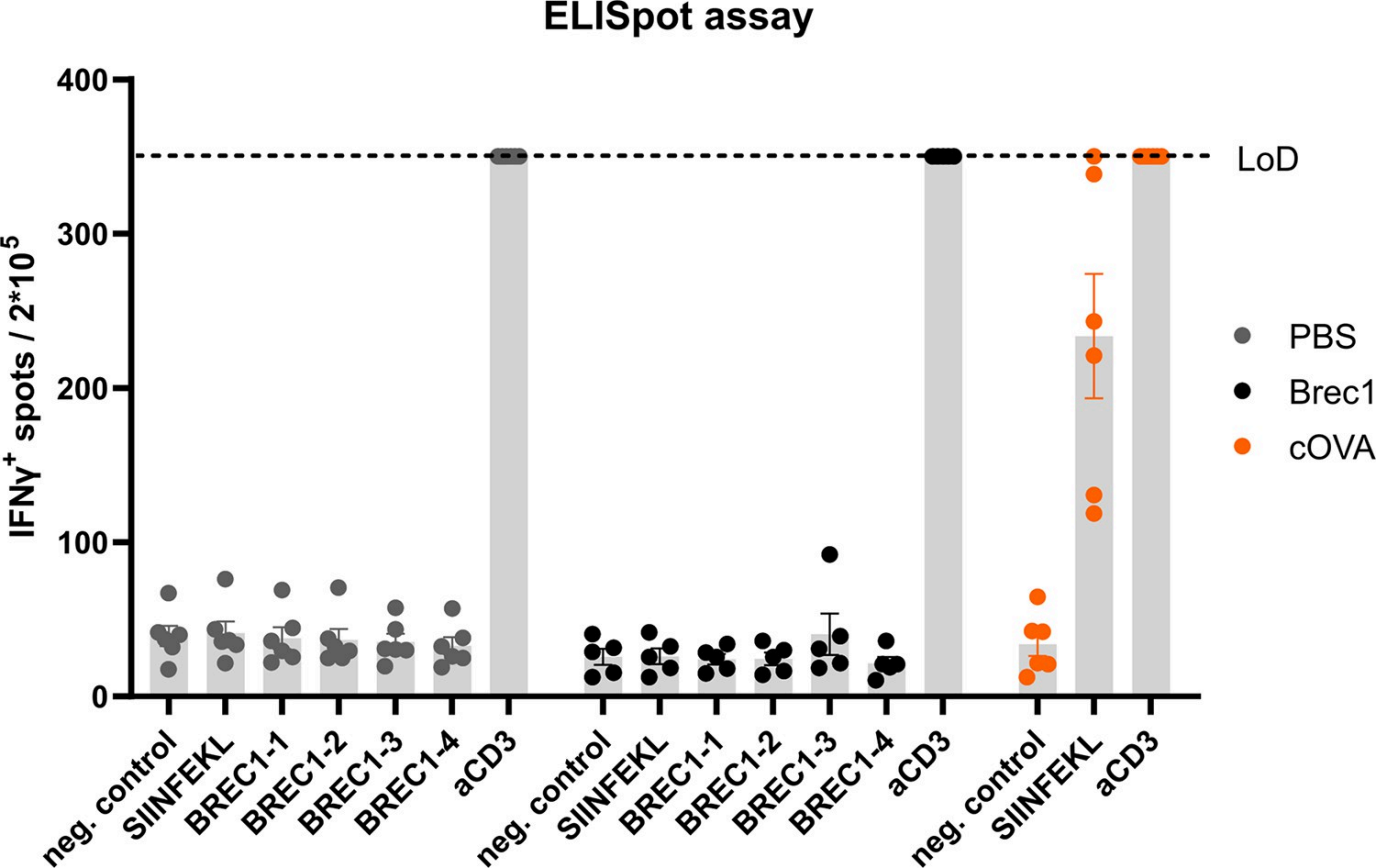
Analysis of Brec1 immunogenicity. C57BL/6 mice were infected with 5–8×10^8^ IU lentiviral vector expressing either Brec1 (black dots), cOVA (non-secreted ovalbumin; orange dots), or with PBS (grey dots). After 28 days, sera and spleens were collected. Spleen cells (2×10^5^/well) were incubated without stimulation (control), with OVA_257-264_ peptide (SIINFEKL), with four pools of Brec1 peptides or with anti-CD3 mAb, and cells secreting IFN-γ were determined using ELISpot assays. The maximal number (limit of detection, LoD) was 350 spots/well; N = 5–6 mice per group.

When IFN-γ production was analyzed by ELISpot, using a Brec1 peptide pool (spanning the entire amino acid sequence of Brec1), the resulting data demonstrated only comparable background signals in the case of the PBS negative control (grey dots) and Brec1-infected spleens (black dots) (Fig 12). In contrast, IFN-γ production was easily observed when cOVA (positive control) was expressed as an antigen (orange dots).

Taken together, these analyses demonstrated the absence of Brec1-induced CD4+ or CD8+ T cell-related immunogenicity in this established experimental setup.

## Discussion

Advanced genome editing technologies potentially allow the development of novel antiviral therapies aiming at antiretroviral drug-free long-term remission of HIV, or even virus eradication [13, 14, 18, 19]. It is anticipated that such new curative concepts will prevent long-term ART-related side effects (i.e. toxicities) [5, 6, 10], ease social stigma frequently experienced by PLWH [74, 75], and, hopefully, also lower current lifetime ART costs [76, 77], thereby significantly reducing the financial burden on public health care systems.

Currently, the major genome editing strategy to interfere with HIV is based on the knock-out of the cellular gene encoding the cell surface CCR5 chemokine receptor that is mostly, but, unfortunately, not exclusively exploited by HIV during *de novo* infection [78, 79]. As observed previously, this non-exclusivity may pose the clinical risk that CCR5 inactivation can result, for example, in the selection of primary CXCR4-tropic virus isolates *in vivo* [80]. Moreover, CCR5 knock-out has been discussed as a risk factor for impaired immune responses in other viral diseases [81], most prominently demonstrated in the case of West Nile Virus (WNV) infection [82, 83].

Alternatively, a more direct antiviral approach would involve strategies to inactivate or excise integrated HIV genomes (proviruses), thereby literally removing the molecular basis of the disease [18–21]. It has been shown that RNA-guided CRISPR/Cas nucleases are not only able to inactivate isolated provirus genes by introducing small nucleotide insertions or deletions (indels), but also can excise almost the entire proviral DNA via recombination of guide RNA (gRNA)-targeted terminal proviral sites [36]. However, Cas, like all nuclease-based technologies, relies not only on the induction of double-strand breaks (DSBs) for targeted DNA manipulation but also on cellular DNA repair [84, 85]. Thus, nucleases (such as CRISPR/Cas) are error-prone, preferentially and uncontrollably introducing indels at target sites, which together with the formation of DSBs, raises concerns about their general safety in the clinical context [86, 87]. For example, and with respect to HIV, the rapid emergence of viral escape mutants has indeed been reported in antiviral CRISPR/Cas strategies [88, 89]. Thus, CRISPR-based DNA base-editing and prime-editing technologies that do not introduce DSBs may be increasingly used in future therapies [90]. Nevertheless, these technologies still rely on cellular DNA repair pathways and are therefore equally error-prone. Moreover, these technologies do not allow the complete removal of the provirus and only allow punctual changes in the genome.

In contrast to the technologies above, site-specific recombinases (SSRs) accurately, predictably, and efficiently modify DNA genomes without generating DSBs or recruiting the cell’s repair pathways [17]. Hence, they do not induce indels and work with nucleotide precision in an error-free manner allowing one to remove almost the entire HIV genome (the provirus) without the emergence of escape variants [41, 42]. Therefore, SSRs such as Brec1 would be highly useful components of future HIV eradication strategies, particularly with respect to clinical *in vivo* application.

Regardless of whether designer nucleases or designer recombinases are developed for therapeutic genome editing, the enzyme coding sequence needs to be delivered into target cells, usually using viral vector systems. Thus, preclinical analysis and subsequent clinical trial application (CTA) requires an in-depth analysis of potential toxicities, not only of the therapeutic transgene (i.e. the nuclease or recombinase), but also of the vector system used.

For initial clinical trials it is conceived that the HIV-specific SSR Brec1 vector will be introduced into either CD4^+^ T cells or CD34^+^ HSC of PLWH. In either case, the Brec1 coding sequence will be delivered by a lentiviral vector (Fig 1). Of note, over previous years a lentiviral SIN vector design has emerged as a benchmark for gene therapy particularly of hematolymphoid cells [91–94].

Here, to investigate the potential off-target effects of Brec1 on the human genome we first analyzed genomic sequences (HGS; Table 2), which were previously identified by computational analysis of the human genome [42]. HGS sites resembled the HIV LTR-specific Brec1 target site *loxBTR* and were previously functionally analyzed in *E*. *coli*, demonstrating the absence of Brec1 activity on these sequences [42]. Here, we now performed locus-specific capture sequencing on these HGS sites and also included potential Brec1 pseudo sites (BTR-off; Table 2), which have since been identified using an *in vitro* assay system [59]. The combined capture sequencing data (Fig 2B and Table 3) demonstrated that Brec1 expression does not genetically alter human endogenous genomic sequences with partial homology to the native viral Brec1 target site *loxBTR*. It is important to note that this analysis was designed to closely mimic the clinical situation by employing LV-Brec1 for long-term transgene expression in human HIV-1 infected T cells (Fig 2). This may explain some discrepancies observed previously for the analyzed BTR-off sites [59]. In that previous study, recombinase activities were functionally analyzed short-term by transient transfection of HEK293T cells using CMV vectors and artificial target sequence reporter plasmids [59].

Finally, the observed absence of Brec1 off-target activity was further confirmed by analyzing the potential recombination of distal genomic Brec1 target sites (Fig 3). In agreement with previously published chromosomal analyses [42], our experiments performed in PM1 T cells again demonstrated the lack of any Brec1-mediated chromosomal rearrangements (Fig 4). These results were also confirmed in HIV-1 infected primary CD4^+^ T cells (Figs 5 and 6). The combined data demonstrated that extended expression of Brec1 in human cells does not result in any detectable alteration (i.e. off-target modification or recombination) in human genomic sequences, which is in complete agreement with previous whole genome NGS data [42].

Our next series of experiments focused on vector toxicity by first analyzing the therapeutic vector LV-Brec1 with respect to its effective VCN, which, when kept low, has been shown to minimize the risk of malignant target cell transformation induced by high numbers of vector insertions [61, 62]. A MOI of 1, which, as recommended by the FDA [65], ensured a total VCN of <5, also resulted in stable Brec1 expression in the absence of any detectable cytotoxicity (Figs 7 and 8).

We then analyzed the potential of LV-Brec1 to directly induce insertional mutagenesis by first employing the well-established IVIM assay, which is based on determining the replating capacity of lentiviral vector-transduced primary murine bone marrow cells [50]. This analysis demonstrated that LV-Brec1 does not induce aberrant cell growth by insertional mutagenesis (Fig 9). Next, we analyzed potential vector genotoxicity by the more sensitive current state-of-the-art SAGA assay, which scores the dysregulation of genes involved in cell immortalization mediated by the vector to be analyzed [54, 69]. These results demonstrated that LV-Brec1 did not dysregulate the oncogenic SAGA gene set (Fig 10). The combined data demonstrate that LV-Brec1 poses a negligible mutagenic risk due to any vector-induced genotoxicity.

We then determined the influence of vector transduction on CD34^+^ PBSC using CFU assays. Using cell samples from two donors, we observed no negative influence of LV-Brec1 on the differentiation potential of human PBSC (Fig 11).

Designer enzymes used for genome editing approaches frequently originate from human pathogens, a fact that may negatively impact the clinical development of novel therapies. For example, various studies described the prevalence of pre-existing anti-Cas antibodies as well as Cas-reactive T cell responses in humans [95–98]. Therefore, we also analyzed the potential of Brec1, which is derived from the bacteriophage P1 Cre enzyme [17], to act as a neo-antigen inducing unwanted and possibly harmful T cell-related immune reactions, such as inflammatory cytokine level-related cytokine storm [99–101] However, infection of test animals with a lentiviral vector [73] constitutively expressing Brec1 followed by ELISpot analysis using Brec1-specific peptide pools revealed only background IFN-γ signals (Fig 12), indicating the absence of Brec1-induced specific CD4+ or CD8+ T cell immunity *in vivo*. Due to the intracellular expression of the protein, we did not expect the formation of an antibody response. In addition, we consider such a response irrelevant because it is unlikely that such antibodies would recognize or interfere with the function of T cells. Therefore, analysis of a potential antibody response was not in the focus of in the present study. However, in the upcoming clinical trial the detailed measurement of all potential immune responses will be addressed in detail.

In summary, our data demonstrate that neither the HIV-specific Brec1 recombinase protein nor the clinical vector LV-Brec1 induces any detectable toxicity, as seen by a complete lack of cytotoxicity, genotoxicity or T cell based immunogenicity in our experiments. This lack of toxicity is in full agreement with previous analyses that included genome-wide NGS, array-comparative genomic hybridization (array-CGH), spectral karyotyping (SKY), as well as assays for cell cycle, apoptosis, and cytokine release from primary CD4^+^ T cells [42]. Therefore, Brec1 appears to be a valuable component of a novel gene therapy strategy allowing safe, controllable, and accurate (i.e. error-free) excision of HIV proviral DNA, and may, therefore, be a fundamental tool to transform HIV infection into a curable disease.

## Supporting information

S1 Table Target regions of potential human genomic Brec1 off-target sites. a: genomic location (human reference genome hg38) of the target region. b: genomic region covered by enrichment capture probes.
(DOCX)

S2 Table Brec1 peptide pools. The Brec1 sequence of 351 amino acids was synthesized as 17-mers with 7 overlapping amino acids (peptides & elephants GmbH) and organized into four pools. *peptide #32 does not exist.
(DOCX)

S3 Table Individual SAGA scores. The table lists the vector, transduction replicate, the normalized enrichment score (NES), and the final gene set enrichment (GSEA) prediction (transforming/untransforming).
(DOCX)

S1 Fig Gene browser views of sequenced genomic DNA fragments. Sequences were aligned in the vicinity of potential off-target sites as indicated by Bessen and coworkers [59]. Each segment represents a DNA fragment identified by a pair of sequencing reads. Colors indicate their alignment quality, with low values corresponding to higher mapping ambiguity. Grey bars indicate the positions of predicted off-target sites. The plots were generated based on whole genome sequencing data from LV-Brec1-GFP-infected PLWH-derived primary CD4^**+**^ T cells [42]. Sequence alignments were performed with bowtie2 default paired-end sequencing pipeline, no read filtering was applied.
(TIF)

S2 Fig Sequencing reads aligned to a random region on chromosome 1. The plots show alignments of reads to a random locus from chromosome 1. Each read is represented by a line starting with an arrow at its 5’ end. Pairs of reads coming from the same fragment are connected by dashed lines. Pairs that are within a reasonable distance and expected orientation (facing each other), are properly aligned and displayed in grey. All reads whose mates are on different chromosomes are labeled in red. All other reads or pairs, which are aligning discordantly, are displayed in orange.
(TIF)

S3 Fig Transduction efficiency of RSF91 and LV-Brec1 in Lin^-^ BM cells. Primary Lin^**-**^ bone marrow cells of C57BL6 mice were left non-transduced as negative controls (A, mock), transduced twice with RSF91 (B, green bars) or with LV-Brec1 (C, black bars). Each bar represents the VCN following separate virus transductions. The error bar represents the duplicates of the ddPCR measurement per sample. Transduction efficiency was determined 15 days post-transduction by isolation of genomic DNA and subsequently ddPCR analysis with primers specific for PRE and housekeeping gene mRPP30 sequences.
(TIF)

S1 Dataset (CSV)

S2 Dataset (CSV)

S3 Dataset (CSV)

S4 Dataset (XLSX)

S5 Dataset (XLSX)

S6 Dataset (CSV)

S7 Dataset (XLSX)

S8 Dataset (CSV)

S9 Dataset (CSV)

S10 Dataset (CSV)

S11 Dataset (XLS)

S12 Dataset (CSV)

S13 Dataset (CSV)

S14 Dataset (CSV)
