# Supplementary material for: Preclinical toxicity analyses of lentiviral vectors expressing the HIV-1 LTR-specific designer-recombinase Brec1
Source: PLoS One. 2024 Mar 8;19(3):e0298542. doi: 10.1371/journal.pone.0298542 (PMC10923487; doi:10.1371/journal.pone.0298542)
Supplement: S1 Table — a: genomic location (human reference genome hg38) of the target region. b: genomic region covered by enrichment capture probes. (DOCX) [file pone.0298542.s001.docx]

|  | target region^a^ | | | probe region^b^ | |
| --- | --- | --- | --- | --- | --- |
| id | **chr** | **start** | **end** | **start** | **end** |
| HGS1-1 | 1 | 121.169.348 | 121.169.382 | 121.168.345 | 121.170.468 |
| HGS1-2 | 1 | 143.956.994 | 143.957.028 | 143.955.991 | 143.958.114 |
| HGS1-3 | 1 | 145.110.955 | 145.110.989 | 145.109.893 | 145.111.991 |
| HGS1-4 | 1 | 206.187.886 | 206.187.920 | 206.186.883 | 206.188.948 |
| HGS2-1 | 4 | 138.478.068 | 138.478.102 | 138.477.067 | 138.479.057 |
| HGS2-2 | 8 | 65.603.300 | 65.603.334 | 65.602.349 | 65.604.343 |
| HGS3-1 | 17 | 20.833.853 | 20.833.887 | 20.832.845 | 20.834.792 |
| HGS3-2 | 17 | 22.537.726 | 22.537.760 | 22.536.722 | 22.538.674 |
| HGS4-1 | 6 | 57.983.492 | 57.983.526 | 57.982.397 | 57.984.533 |
| HGS4-2 | 6 | 60.734.963 | 60.734.997 | 60.733.867 | 60.736.004 |
| HGS5-1 | 2 | 87.894.679 | 87.894.713 | 87.893.573 | 87.895.724 |
| HGS5-2 | X | 144.143.848 | 144.143.882 | 144.142.836 | 144.144.987 |
| HGS6-1 | 9 | 39.631.897 | 39.631.931 | 39.630.789 | 39.632.909 |
| HGS6-2 | 9 | 42.385.618 | 42.385.652 | 42.384.576 | 42.386.653 |
| HGS6-3 | 9 | 62.710.515 | 62.710.549 | 62.709.491 | 62.711.653 |
| HGS6-4 | 9 | 66.745.043 | 66.745.077 | 66.744.018 | 66.746.078 |
| BTR-off3 | 1 | 159.864.674 | 159.864.708 | 159.863.670 | 159.865.708 |
| BTR-off4 | 3 | 167.733.225 | 167.733.259 | 167.732.169 | 167.732.313 |
| BTR-off6 | 7 | 125.265.273 | 125.265.307 | 125.264.577 | 125.266.312 |
| BTR-off8 | 12 | 103.496.596 | 103.496.003 | 103.495.567 | 103.497.633 |
