# Supplementary material for: Preclinical toxicity analyses of lentiviral vectors expressing the HIV-1 LTR-specific designer-recombinase Brec1
Source: PLoS One. 2024 Mar 8;19(3):e0298542. doi: 10.1371/journal.pone.0298542 (PMC10923487; doi:10.1371/journal.pone.0298542)
Supplement: S2 Table — The Brec1 sequence of 351 amino acids was synthesized as 17-mers with 7 overlapping amino acids (peptides & elephants GmbH) and organized into four pools. *peptide #32 does not exist. (DOCX) [file pone.0298542.s002.docx]

| **# peptide pool** | **Brec1 peptides*** |
| --- | --- |
| 1 | 1-9 |
| 2 | 10-18 |
| 3 | 19-26 |
| 4 | 27-35 |
