# Supplementary material for: Preclinical toxicity analyses of lentiviral vectors expressing the HIV-1 LTR-specific designer-recombinase Brec1
Source: PLoS One. 2024 Mar 8;19(3):e0298542. doi: 10.1371/journal.pone.0298542 (PMC10923487; doi:10.1371/journal.pone.0298542)
Supplement: S3 Table — The table lists the vector, transduction replicate, the normalized enrichment score (NES), and the final gene set enrichment (GSEA) prediction (transforming/untransforming). (DOCX) [file pone.0298542.s003.docx]

| **Vector** | **Replicate** | **NES (GSEA)** | **Prediction.GSEA** |
| --- | --- | --- | --- |
| RSF91 | 1 | 2,24 | transforming |
| RSF91 | 6 | 1,83 | transforming |
| RSF91 | 9 | 1,41 | transforming |
| RSF91 | 2 | 1,58 | transforming |
| RSF91 | 3 | 1,40 | transforming |
| RSF91 | 4 | 1,74 | transforming |
| RSF91 | 5 | 1,67 | transforming |
| RSF91 | 7 | 1,60 | transforming |
| RSF91 | 8 | 1,48 | transforming |
| RSF91 | 10 | **0,93** | untransforming |
| RSF91 | 11 | **-0,86** | untransforming |
| RSF91 | 12 | 1,34 | transforming |
| LV-Brec | 16 | **-1,31** | untransforming |
| LV-Brec | 10 | **-1,53** | untransforming |
| LV-Brec | 1 | **-1,50** | untransforming |
| LV-Brec | 2 | **-1,62** | untransforming |
| LV-Brec | 13 | **-1,01** | untransforming |
| LV-Brec | 19 | **-1,69** | untransforming |
| LV-Brec | 4 | **-1,65** | untransforming |
| LV-Brec | 14 | **-1,59** | untransforming |
| LV-Brec | 9 | **-1,52** | untransforming |
| LV-Brec | 8 | **-1,54** | untransforming |
| LV-Brec | 18 | **-1,47** | untransforming |
| LV-Brec | 23 | **-1,70** | untransforming |
| LV-Brec | 15 | **-1,37** | untransforming |
| LV-Brec | 11 | **-1,81** | untransforming |
| LV-Brec | 3 | **-1,57** | untransforming |
| LV-Brec | 22 | **-1,47** | untransforming |
| LV-Brec | 12 | **-1,73** | untransforming |
| LV-Brec | 6 | **-1,77** | untransforming |
| LV-Brec | 21 | **-1,72** | untransforming |
| LV-Brec | 17 | **-1,57** | untransforming |
| LV-Brec | 20 | **-1,67** | untransforming |
| LV-Brec | 24 | **-1,62** | untransforming |
