# Supplementary figures and images for: Preclinical toxicity analyses of lentiviral vectors expressing the HIV-1 LTR-specific designer-recombinase Brec1
Source: PLoS One. 2024 Mar 8;19(3):e0298542. doi: 10.1371/journal.pone.0298542 (PMC10923487; doi:10.1371/journal.pone.0298542)

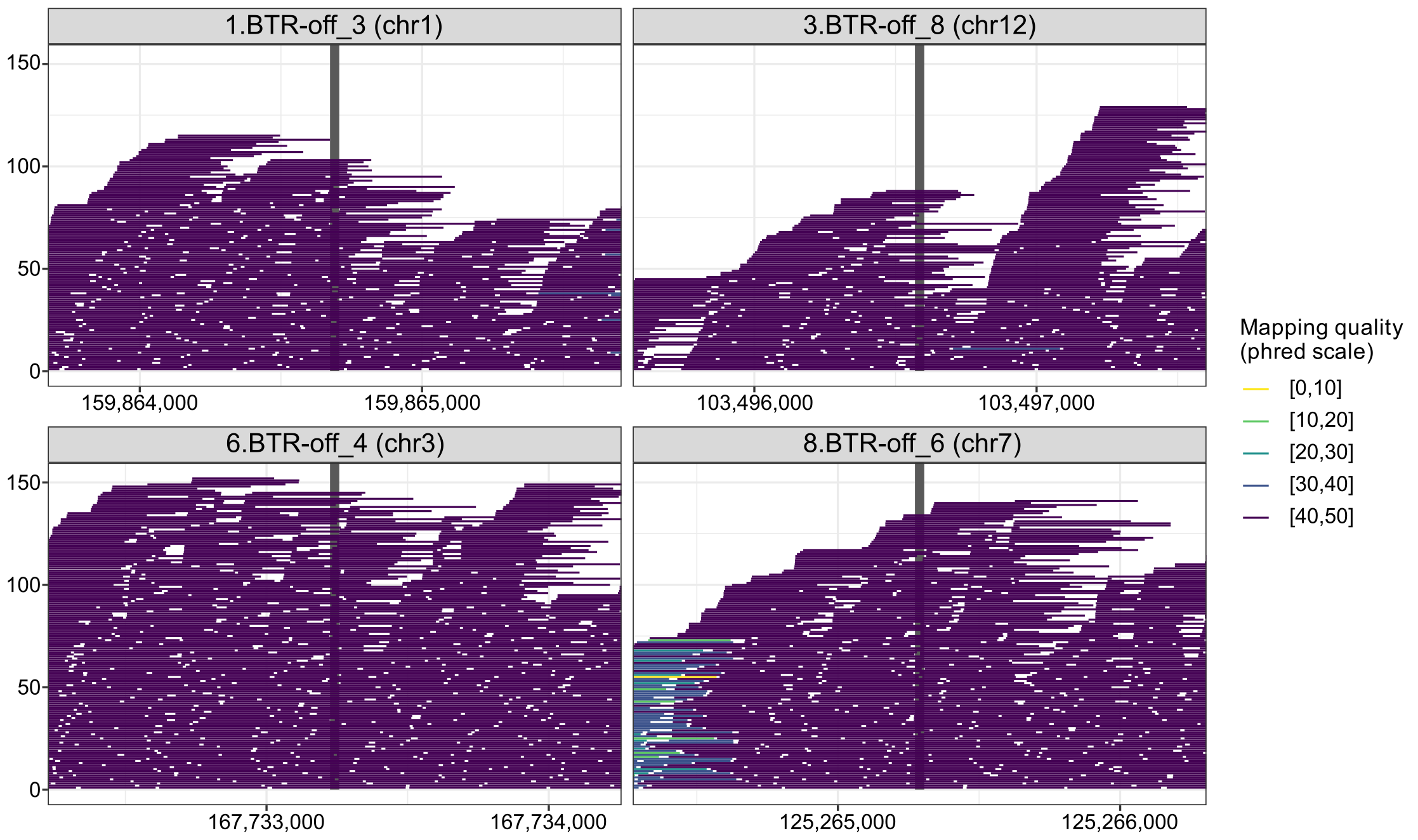

Supplement: S1 Fig — Sequences were aligned in the vicinity of potential off-target sites as indicated by Bessen and coworkers [59]. Each segment represents a DNA fragment identified by a pair of sequencing reads. Colors indicate their alignment quality, with low values corresponding to higher mapping ambiguity. Grey bars indicate the positions of predicted off-target sites. The plots were generated based on whole genome sequencing data from LV-Brec1-GFP-infected PLWH-derived primary CD4+ T cells [42]. Sequence alignments were performed with bowtie2 default paired-end sequencing pipeline, no read filtering was applied. (TIF) [file pone.0298542.s004.tif]

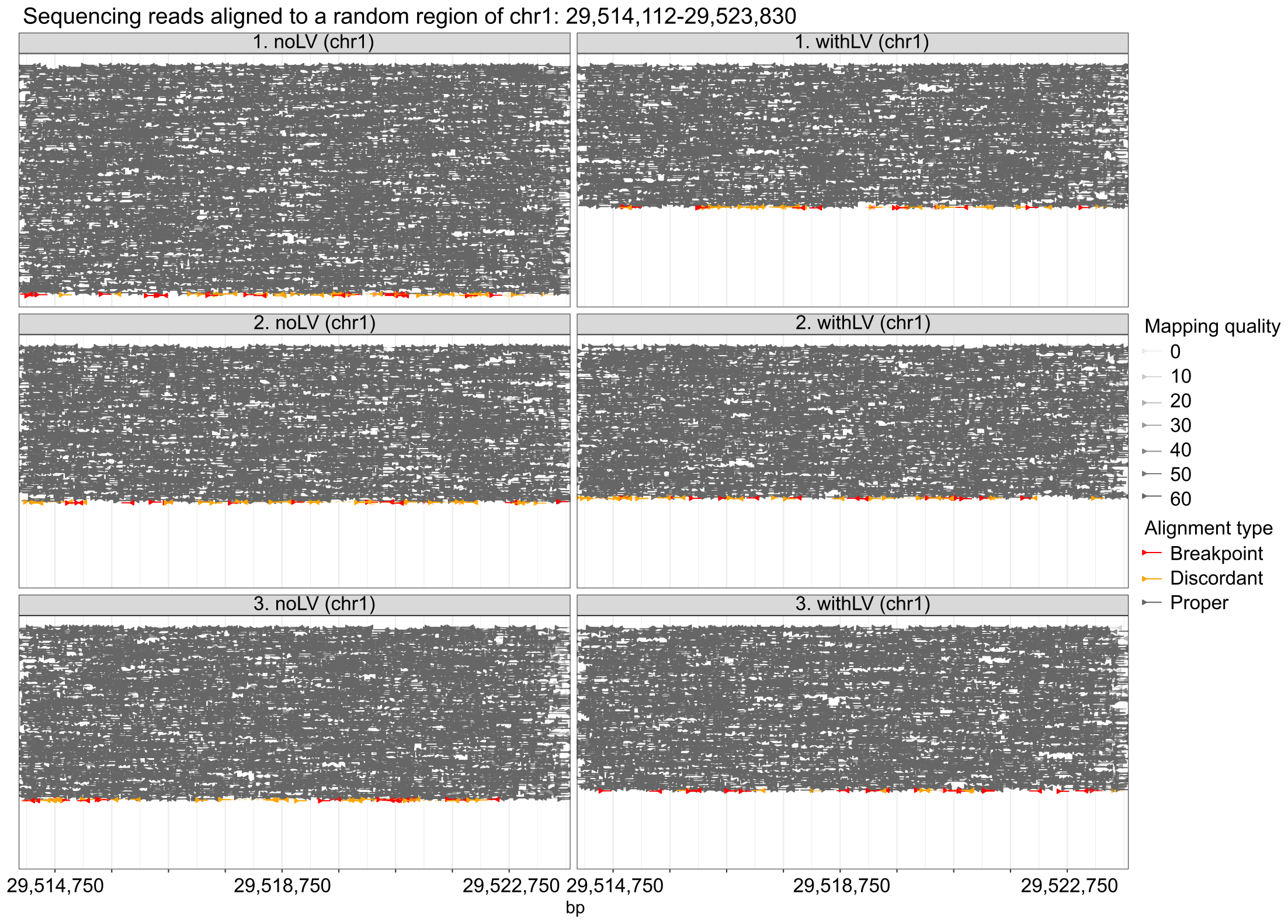

Supplement: S2 Fig — The plots show alignments of reads to a random locus from chromosome 1. Each read is represented by a line starting with an arrow at its 5’ end. Pairs of reads coming from the same fragment are connected by dashed lines. Pairs that are within a reasonable distance and expected orientation (facing each other), are properly aligned and displayed in grey. All reads whose mates are on different chromosomes are labeled in red. All other reads or pairs, which are aligning discordantly, are displayed in orange. (TIF) [file pone.0298542.s005.tif]

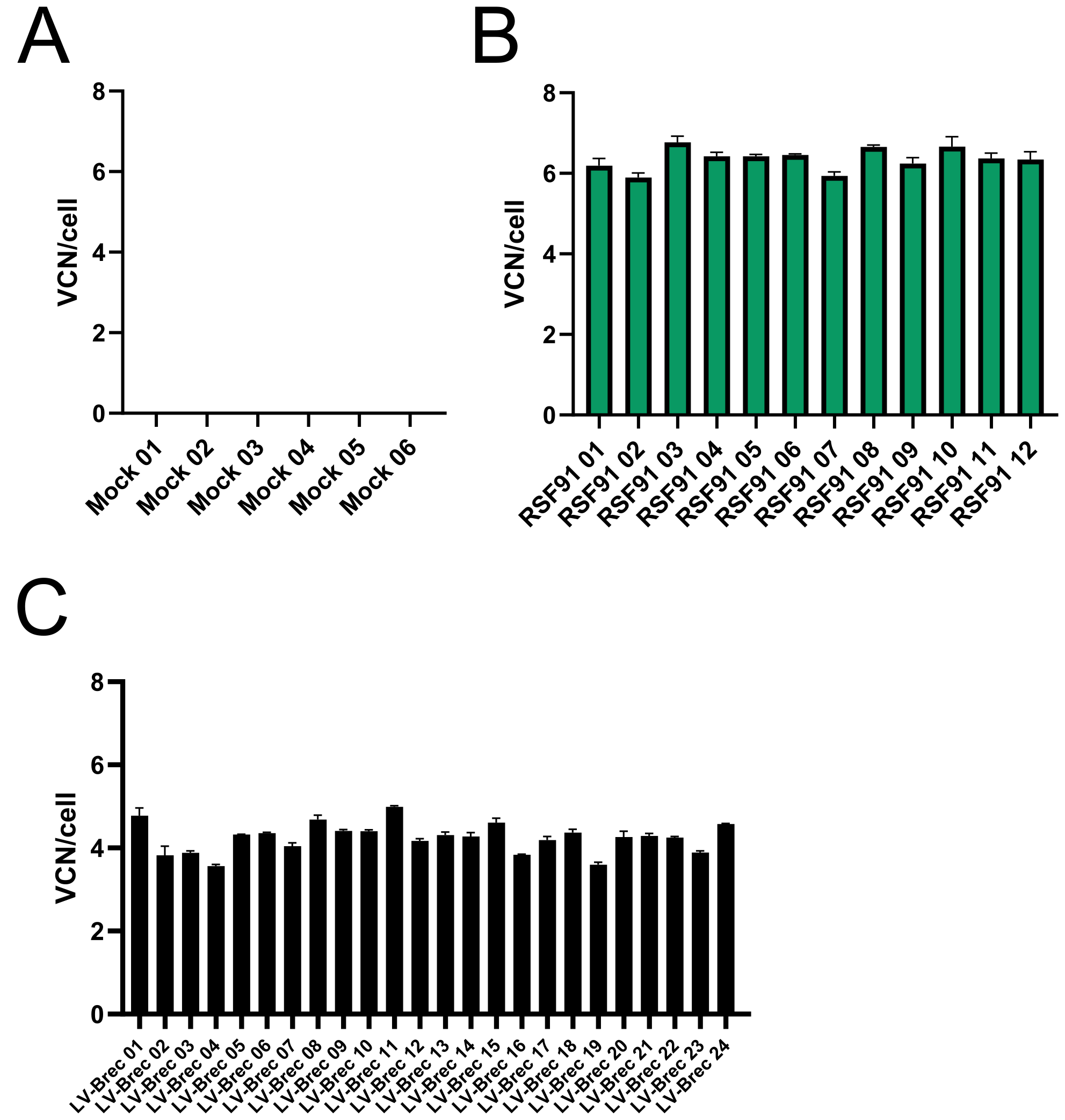

Supplement: S3 Fig — Primary Lin- bone marrow cells of C57BL6 mice were left non-transduced as negative controls (A, mock), transduced twice with RSF91 (B, green bars) or with LV-Brec1 (C, black bars). Each bar represents the VCN following separate virus transductions. The error bar represents the duplicates of the ddPCR measurement per sample. Transduction efficiency was determined 15 days post-transduction by isolation of genomic DNA and subsequently ddPCR analysis with primers specific for PRE and housekeeping gene mRPP30 sequences. (TIF) [file pone.0298542.s006.tif]
